# High resolution melting: improvements in the genetic diagnosis of hypertrophic cardiomyopathy in a Portuguese cohort

**DOI:** 10.1186/1471-2350-13-17

**Published:** 2012-03-19

**Authors:** Susana Santos, Vanda Marques, Marina Pires, Leonor Silveira, Helena Oliveira, Vasco Lança, Dulce Brito, Hugo Madeira, J Fonseca Esteves, António Freitas, Isabel M Carreira, Isabel M Gaspar, Carolino Monteiro, Alexandra R Fernandes

**Affiliations:** 1 Centro de Química Estrutural, Instituto Superior Técnico, Technical University of Lisbon, Lisbon, Portugal; 2 Faculdade de Engenharia e Ciências Naturais, Universidade Lusófona de Humanidades e Tecnologias, Lisbon, Portugal; 3 Faculdade de Farmácia, University of Lisbon, Lisbon, Portugal; 4 CENCIFOR - Forensic Sciences Centre, Coimbra, Portugal; 5 Centro de Metabolismo e Endocrinologia, Faculty of Medicine, University of Lisbon, Lisbon, Portugal; 6 Centro de Cardiologia, Faculty of Medicine, University of Lisbon, Lisbon, Portugal; 7 Centro de Medicina Desportiva de Lisboa, Lisboa, Portugal; 8 Laboratory of Cytogenetics and Genomics, Faculty of Medicine, University of Coimbra, Coimbra, Portugal; 9 Genética Médica, Hospital Egas Moniz, Baltimore, MD, USA; 10 Cardiogenética, Hospital Santa Cruz, Centro Hospitalar Lisboa Ocidental, Lisboa, Portugal; 11 Departamento Ciências da Vida, Faculty of Scienses and Technologies, Universidade Nova de Lisboa, Lisboa, Portugal

**Keywords:** Hypertrophic cardiomyopathy, Gene-based diagnosis, High Resolution Melting, Sarcomere proteins, *CSRP3 *gene

## Abstract

**Background:**

Hypertrophic Cardiomyopathy (HCM) is a complex myocardial disorder with a recognized genetic heterogeneity. The elevated number of genes and mutations involved in HCM limits a gene-based diagnosis that should be considered of most importance for basic research and clinical medicine.

**Methodology:**

In this report, we evaluated High Resolution Melting (HRM) robustness, regarding HCM genetic testing, by means of analyzing 28 HCM-associated genes, including the most frequent 4 HCM-associated sarcomere genes, as well as 24 genes with lower reported HCM-phenotype association. We analyzed 80 Portuguese individuals with clinical phenotype of HCM allowing simultaneously a better characterization of this disease in the Portuguese population.

**Results:**

HRM technology allowed us to identify 60 mutated alleles in 72 HCM patients: 49 missense mutations, 3 nonsense mutations, one 1-bp deletion, one 5-bp deletion, one in frame 3-bp deletion, one insertion/deletion, 3 splice mutations, one 5'UTR mutation in *MYH7*, *MYBPC3*, *TNNT2*, *TNNI3*, *CSRP3*, *MYH6 *and *MYL2 *genes. Significantly 22 are novel gene mutations.

**Conclusions:**

HRM was proven to be a technique with high sensitivity and a low false positive ratio allowing a rapid, innovative and low cost genotyping of HCM. In a short return, HRM as a gene scanning technique could be a cost-effective gene-based diagnosis for an accurate HCM genetic diagnosis and hopefully providing new insights into genotype/phenotype correlations.

## Background

Hypertrophic cardiomyopathy (HCM) is the most common monogenic disease of the cardiovascular trait affecting 1:500 individuals in the general population [1-3]. HCM is classically characterized by unexplained left ventricular hypertrophy (LVH) and distinct histopathological features comprising myocyte disarray and interstitial fibrosis [1-3]. Clinical evaluation may be triggered in response to symptoms, in asymptomatic individuals in the course of a family screening, or after detection of a systolic murmur or an abnormal electrocardiogram (ECG). HCM diagnosis is typically performed by the identification of unexplained LVH on cardiac imaging studies. Nevertheless, the morphologic pattern of LVH is not closely predictive of the severity of symptoms or prognosis [1]. HCM is a complex and heterogeneous disease with remarkable diversity in the course of disease, age of onset, symptoms severity, left ventricular outflow obstruction, and risk for sudden cardiac death (SCD) [1]. Indeed, some individuals experience no or only minor symptoms, while others may develop refractory symptoms or end stage heart failure (HF) requiring cardiac transplantation. The most and serious consequences of HCM are though, HF and SCD [1,2,4,5]. HCM is also the leading cause of SCD in competitive athletes in whom it may be the only clinical manifestation of the disease [6].

Genetic studies established the paradigm that HCM is a disease of the sarcomere. With a dominant transmission pattern, HCM is caused by over 1000 distinct mutations identified in over 29 genes including those encoding sarcomeric proteins (Table 1) [1]. This fact underlines that behind the clinical heterogeneity seen in HCM there is also substantial genetic heterogeneity, at both allelic and non-allelic levels. With a higher prevalence when family history of HCM is present, mutations in the genes encoding cardiac b-myosin heavy chain (*MYH7*), cardiac myosin binding protein C (*MYBPC3*), cardiac troponin T (*TNNT2*), and cardiac troponin I (*TNNI3*) are the most prevalent and all together account for over 80%-90% of HCM cases [1,7,8].

**Table 1:** Proteins/genes associated with HCM

Protein	Gene	OMIM ID	Reference sequence^(a)^	Mutations Known^(b)^/prevalence^(c)^
**Sarcomeric Proteins**				
Cardiac myosin-binding protein C^(d)^	*MYBPC3 *	600958	NM_000256	294/~40
β-cardiac myosin heavy chain^(d)^	*MYH7 *	160760	NM_000257	335/~40
Cardiac Troponin T^(d)^	*TNNT2 *	191045	NM_000364	60/~5
Cardiac Troponin I^(d)^	*TNNI3 *	191044	NM_000363	50/~5
Regulatory myosin light chain	*MYL2 *	160781	NM_000432	13/~1
α-Tropomyosin^(d)^	*TPM1 *	191010	NM_0001018005	24/~2
Cardiac actin	*ACTC1 *	102540	NM_005159	18/~1
Essential myosin light chain	*MYL3 *	160790	NM_000258	11/~1
Cardiac Troponin C	*TNNC1 *	191040	NM_003280	12/rare
α-myosin heavy chain	*MYH6 *	160710	NM_002471	22/rare
Titin	*TTN *	188840	NM_133378	20/rare
Obscurin	*OBSCN *	608616	NM_052843	1/rare
Desmin	*DES *	125660	NM_001927	1/rare
Caveolin 3	*CAV3 *	601253	NM_033337	1/rare
**Z-band**				
Muscle LIM protein (MLP)	*CSRP3 *	600824	NM_003476	12/?
Telethonin	*TCAP *	604488	NM_003673	11/?
Myozenin 2	*MYOZ2 *	605602	NM_016599	2/?
Vinculin	*VCL *	193065	NM_014000	1/?
**Calcium-Handling HCM**				
Junctophilin 2	*JPH2 *	605267	NM_020433	4/?
Phospholamban	*PLN *	172405	NM_002887	8/?
Myosin Light Chain Kinase 2	*MYLK2 *	606566	NM_033118	2/?
Calsequestrin 2	*CASQ2 *	114251	NM_001232	1/?
**Other non-sarcomeric proteins**				
Myosin VI	*MYO6 *	600970	NM_004999	1/?
Solute carrier family 25 member 4	*SLC25A4 *	103220	NM_001151	1/?
Cytochrome C oxidase assembly protein 15	*COX15 *	603646	NM_078476	2/?
**HCM Phenocopies**				
2 subunit of AMP-activated protein kinase	*PRKAG2 *	602743	NM_016203	10/?
V-raf-1 murine leukemia viral oncogene homolog 1	*RAF1 *	164760	NM_002880	1/?
Frataxin	*FXN *	606829	NM_000144	1/?
Lysosome associated membrane 1	*LAMP2 *	309060	NM_002294.2	1/?

These include the sarcomeric proteins, Z-band, calcium-handling, mitochondria, HCM phenocopies and other non-sarcomeric proteins whose mutations have been associated with HCM. The OMIM, Reference sequence^(a)^, number of HCM-associated mutations known^(b) ^and prevalence of mutations^(c) ^are also indicated

^(a) ^UCSC Genome Browser http://genome.ucsc.edu/cgi-bin/hgGateway; ^(b) ^Human Genome Mutation Database http://www.hgmd.org and Harvard Sarcomere mutation Database http://genepath.med.harvard.edu/~seidman/cg3/;^(c) ^[1]; ^(d) ^Clinical Genetic Test available in Portugal

Although rare, mutations in other sarcomere- associated genes, particularly in Z-band proteins coding genes such as telethonin (*TCAP*) and muscle LIM protein (*CSRP3*), have been described in HCM (Table 1) [9]. The Z band is critical for cyto-architecture and is involved in mechanosensory signalling of cardiomyocytes [9]. Proteins involved in calcium-induced release and signalling and in mitochondrial function have also been implicated in HCM (Table 1) [1,10-12]. Phenocopies of HCM have been identified were cardiac hypertrophy is caused by mutations in genes distinct from those that encode sarcomere proteins (Table 1) [1].

Currently, most of the mutations identified are novel sequence variants, not previously identified as pathogenic mutations [1,13-16]. Furthermore, when novel sequence variants are identified, family studies to confirm cosegregation of the mutation are valuable. A relatively high rate (3% to 10%) of compound heterozygosity has also been observed in HCM [1,14,17,18].

Recent genetic studies demonstrate variability in the types of HCM-mutations among different populations. In the Netherlands or in the Southeast Asian Indian population HCM-founding mutations are the main cause while in the USA there is marked genetic heterogeneity and most HCM patients have a unique pathogenic mutation [1,19,20]. However, HCM genetic basis in Portugal has not yet been extensively studied [4-6,21]. Understanding the genetic basis of HCM provides an opportunity for gene-based diagnosis with benefits for HCM in basic research and clinical medicine, which are currently limited by the considerable costs of genetic testing strategies and an incomplete knowledge of all disease genes [1,8,10,13,22].

The current "gold standard" genetic diagnosis test for HCM in Portugal is automated dideoxi-Sequencing (AS) of the most frequent mutated HCM-genes (Table 1-^(d)^). AS has a low throughput and high cost, and most importantly it does not include genes coding for other proteins related to myocardium contraction such as the Z-band. As a consequence more than 1/3 of proband remains to characterize as no mutation is found. This fact underlines the need to screen more genes using high-throughput sensitive techniques which will enable a more reliable genetic diagnosis [23,24] and further hampers the cost effectiveness of the current genetic test.

In fact, the number of HCM phenotype-associated mutations in 29 different genes (Table 1), make full molecular testing by AS unbearably slow and expensive, prompting Real time PCR techniques has an alternative technique [18,24]. High Resolution Melting (HRM) is a high throughput gene variation scanning technique that relies on the differential melting properties of sequences that vary in at least one nucleotide. For this reason it is used to identify novel mutations within samples. Unlike other scanning methods, HRM analysis provides a closed tube system that reduces the risk of contamination, decreases analysis time and requiring no post-PCR sample handling. Recent results from our group [24] and others [18] have already proved the effectiveness of this technique for the identification of alterations in HCM patients. Nevertheless, the cohorts of population used in both studies were low (13 and 34 respectively) [18,24].

The aim of the present work was to improve HCM gene-based diagnosis by applying a high throughput Real Time PCR-based technique, HRM, to study the prevalence of HCM-mutations in a higher Portuguese HCM cohort. The results of this work will contribute to understand the genetic basis and variability in HCM among our population.

## Population and methods

### DNA samples

Peripheral blood was collected from 80 Portuguese unrelated patients and from 100 healthy subjects (controls without any cardiovascular disease). With the exception of control individuals, all index patients presented either familial history of HCM or clinical diagnosis of HCM. Diagnosis was established on clinical, ECG and Echocardiograph (ECHO) grounds. ECG/ECHO criteria have been previously discussed and published [5]. Available biometrical characteristics and clinical data are described in Table 2. This study was approved by the Ethics Committee of the Lisbon Faculty of Medicine and all patients have signed an informed consent for genetic analysis. DNA was extracted from the blood samples using a Blood DNA kit (Roche Applied Science) accordingly with the manufacturer procedure http://www-roche.com.

**Table 2:** Biometric and clinical data of HCM patients studied in this work

Patient sample code	Gender: Male (M), Female (F)	Age HCM:familial (f), sporadic (s)	Hypertrophy: apical (a), septal (s), diffuse (d), parietal (p), obstructive (ob)	CDI:Yes (y), No (n)	SCD familial history:Yes (y), No (n)
**1**	F	55 f	d (> s), ob, deceased	y	y
**2**	M	19 s	na	na	na
**3**	M	18 f	s	n	y
**4**	F	68 s	p	n	n
**5**	M	69 s	p, s	n	n
**6**	M	50 s	na	na	na
**7**	M	25 s	d (> s), ob	n	n
**8**	M	58 f	d	n	na
**9**	M	72 f	a	n	na
**10**	M	na f	d (> s)	n	n
**11**	M	33 f	s	n	n
**12**	M	37 f	na	na	na
**13**	M	58 f	a	y	na
**14**	M	na f	s	n	n
**15**	M	58 f	na	n	na
**16**	M	38 f	a	y	na
**17**	F	61 f	a	n	na
**18**	M	51 s	s, ob	n	n
**19**	F	64 s	na	n	na
**20**	F	32 s	na	na	na
**21**	F	69 f	na	na	na
**22**	M	55 f	na	na	na
**23**	M	na s	na	na	na
**24**	M	22 f	s	n	n
**25**	F	25 s	na	na	na
**26**	F	31 s	s	n	n
**27**	M	na f	s	n	n
**28**	F	62 f	a	y	n
**29**	M	19 s	na	na	n
**30**	F	55 f	a	y	na
**31**	M	54 f	a	n	na
**32**	F	61 s	p	n	n
**33**	M	51 f	na	na	na
**34**	M	39 f	s	y	na
**35**	M	19 s	na	na	na
**36**	M	42 s	na	na	na
**37**	M	29 s	s	na	na
**38**	M	30 f	na	na	na
**39**	M	67 f	s	y	y
**40**	M	21 s	d	n	n
**41**	F	na s	p, s, ob	n	n
**42**	F	70 s	na	na	na
**43**	F	14 s	na	na	na
**44**	M	84 f	na	na	na
**45**	F	45 f	na	na	na
**46**	M	42 f	na	na	na
**47**	M	na s	na	na	na
**48**	M	44 s	p	n	n
**49**	M	47 s	p, s, ob	n	n
**50**	M	50 s	na	y	na
**51**	F	82 s	d (> s)	n	n
**52**	F	64 f	na	na	na
**53**	F	74 f	p, s	n	n
**54**	M	59 s	ob	y	na
**55**	M	53 f	na	na	na
**56**	F	70 f	a	n	na
**57**	F	61 f	s, ob	y	y
**58**	M	41 f	na	na	na
**59**	F	18 s	na	na	na
**60**	M	na s	d	na	na
**61**	M	na s	a	n	n
**62**	M	na f	s	n	n
**63**	F	78 s	na	na	na
**64**	M	54 s	na	na	na
**65**	M	82 s	p, s, ob	n	n
**66**	M	na s	na	na	na
**67**	M	21 s	s	na	n
**68**	M	na s	s	n	n
**69**	F	31 s	a	n	n
**70**	F	78 s	p	n	n
**71**	M	52 f	s	n	n
**72**	F	49 f	na	na	na
**73**	F	78 f	a	n	na
**74**	M	42 s	na	na	na
**75**	F	58 f	na	na	na
**76**	F	80 f	na	na	na
**77**	F	78 s	na	na	na
**78**	F	na f	na	na	na
**79**	F	na f	d (> s), ob, deceased	n	n
**80**	M	55 f	na	na	n

For some of the patients no information was available regarding the data analyzed (na: data not available; CDI - cardio defibrillator; SCD - sudden cardiac death)

### HRM analysis

Mutation scanning of all samples was performed using a HRM technique in a Real-Time PCR thermal cycler (LightCycler 480, Roche Diagnostics) as described in our previous work [24]. Primers were constructed according to the common HRM specifications http://www.gene-quantification.de/LC480-Technical-Note-01-HRM.pdf, using the following online software and databases: UCSC Genome Browser http://genome.ucsc.edu/cgi-bin/hgGateway, Primer 3 http://frodo.wi.mit.edu/, DINAMelt http://mfold.rna.albany.edu/?q=DINAMelt, Poland service http://www.biophys.uni-duesseldorf.de/local/POLAND/poland.html and Primer-BLAST http://www.ncbi.nlm.nih.gov/tools/primer-blast (Additional file 1: Table 1). Amplicon lengths were kept relatively short to improve the detection of genetic variations. In this regard, exons which were greater than 315 bp or exons with more than two melting domains were amplified in more than one overlapping segments, covering exons/intron boundaries (Additional file 1: Table 1). The HPLC grade primers were purchased from Metabion (Germany). Exons 3 and 12 of the *MYH7 *gene, exon 30 of the *MYBPC3 *gene and exons 1, 2, and 3 of *TNNI3 *gene contain several highly prevalent polymorphisms, nevertheless, they were also included in our study and whenever an alteration was found in one of those genomic regions by HRM, the corresponding sample was submitted to AS. Reference sequences of the genes were obtained from the Genome Browser of the University of California Santa Cruz http://genome.ucsc.edu/cgi-bin/hgGateway. LightCycler^®^480 High Resolution Melting Master kit (Roche Applied Science) which includes a hot start PCR enzyme and the ResoLight saturating DNA dye was used for the amplification of all the 208 genomic regions, accordingly with the manufacturer procedure http://www.roche.com. The PCR conditions used to amplify all amplicons are described in Additional file 1: Table 1. The amplification conditions were composed of 35 PCR cycles of denaturation at 95°C for 10 seconds, primer annealing at an optimized temperature for 10 seconds and an extension at 72°C for 20 seconds. Melting and fluorescence detection conditions were as indicated by the supplier (Roche Diagnostics). Briefly, immediately after amplification, the PCR products were heated to 95°C and cooled to 40°C (for heteroduplex formation), and melting was monitored (by fluorescence emission) from 65°C to 95°C. Using the LightCycler 480^®^1.5.0.39 SP3 software (Roche Diagnostics), melting data was normalized, temperature shifted and displayed as derivative curves compared to the control samples. The auto-group function was applied to generate automatic genotype group analyzing the melt profiles and clustering samples into groups with similar melting profiles. An altered sample was considered whenever: the generated melting curve was different from control samples; it was grouped apart from the controls; and presented a relative signal difference out of the range of normality limits (+2 to -2. When an altered profile was found, the PCR product was purified and sequenced (as a paid service, TRACEGEN).

Whenever a mutation was confirmed in a proband, first and second degree relatives were tested, whenever possible, for the identified variant in order to check the co-segregation with the disease in the family. Except for non sense and frame shift mutations, due to short insertions or deletions and splice site mutations, a missense variant was considered as a mutation when: - it co-segregates with the disease in the family; - affects an amino acid conserved among species and isoforms; and - it failed to be detected in at least 200 chromosomes of healthy adult controls (CS01-CS101).

### Single-strand conformation polymorphism analysis

In order to confirm HRM reliability, mutation scanning was also performed through Single-Strand Conformation Polymorphism (SSCP) analysis. Selected exons and flanking intronic regions were amplified through PCR. Each amplicon was denatured and single strand conformational profile was evaluated using GenePhor™ DNA Electrophoresis System and PlusOne™ DNA Silver Staining Kit (GE HealthCare Life Sciences). All amplicons with different migration pattern (by comparison with the same amplicon from a healthy control individual) were sequenced for DNA alteration confirmation.

## Results and discussion

### PCR optimization

Since 2002, HRM scanning method has been applied in human diseases genotyping and used for high throughput mutation scanning on genes, for which large cohorts of patients have to be investigated [18,24-29]. As described previously by us and others [18,24], HRM scanning accuracy depends on high quality PCR, meaning that optimization a critical step and that the presence of primer dimers or non specific products may alter the melt curve characteristics causing the generation of false positive results. HRM analysis optimization was previously performed by us, using 3 control DNAs (previously sequenced) and 7 DNAs carrying previously identified HCM genetic variants (see Additional file 1: Table 1). Using this strategy we have demonstrated HRM potential to identify mutations in genes that are not usually analyzed in standard diagnostic techniques such as AS [24].

### Molecular analysis of 80 HCM patients

The fact that the genetic basis for HCM in Portugal has not yet been extensively studied in large cohort of HCM patients, and the success of HRM as a high throughput and fast analysis technology, drove us to perform a mutational screening of 28 HCM-associated genes using this technology in 80 Portuguese HCM patients (not previously studied). This cohort was also studied by SSCP where all genetic variants detected by HRM analysis were also identified. Similar results were previously reported for other genes such as *F8*, *NF2*, *CFTR*, *LMNA*, and *SCN5A *[25-29]. All DNA amplicons detected as altered through HRM or SSCP were sequenced for mutation confirmation. As HRM could induce some false positives, each amplicon was tested against a mean of 3 wild-type samples in order to decrease significantly the number of false positive calls.

Molecular analysis of this cohort allowed the identification of 96 genetic variants, of which 38 were HCM-known mutated alleles (31 missense mutations, three nonsense mutations, one 1-bp deletion, one in frame 3-bp deletion, one 5-bp deletion and one splice mutation) and 22 were novel mutations (18 missense mutations, one indel, two splice mutations and one promoter mutation) (Table 3).

**Table 3:** List of genetic variations identified by HRM analysis

Exon	Mutation^(a) ^or SNP^(b) ^code or reference	Genetic variation	Patient sample code
***MYBPC3***			
4	rs3729986	c.465 G > A, p.Val158Met	1
5	rs11570052	c.565 G > A, p.Val189Ile	2
6	**CM043536**	**c.706 A > G, p.Ser236Gly**	3, 4, 5
7	[24]	**c.817 C > T, p.Arg273Cys**	6
8	New	c.851 + 95 C > G	7
9	CS041890	c.852-20 C > A	2
13	Synonym	c.1233 T > G, p.Phe411Phe	8
14	New	c.1454 + 34 G > C	9
15	**CM981324**	**c.1481 G > A, p.Arg494Gln**	10
	**CM981325**	**c.1502 G > A, p.Arg501Gln**	11, 12, 13
16	[24]	**c.1727 G > A; p.Trp576X**	14
	Synonym	c.1626 G > C, p.Lys542Lys	9, 15
19	**New**	**c.1945insT1941-1946delCCTGGA**,	16, 17
		**p.Pro647fs**	
18	New	c.1925-24 T > C	18
21	rs3729948	c.2305 + 18 C > G	19
22	**New**	**c.2344 G > C, p.Val782Leu**	20
23a	**New**	**c.2470 C > A, p.Leu824Met**	10
	**CM041808**	**c.2523 C > G p.Tyr841X**	21
	**New**	**c.2411-2 A > T**	22
24	rs3729936	c.2734 + 12 C > T	19
	**CM992932**	**c.2683 G > A, p.Val895Met**	23, 24
25	**CM032959**	**c.2824 C > T, p.Arg942X**	1, 25, 26
	[24]	**c.2759A > T, pGln920Leu**	11, 12
	**New**	**c.2780 C > T;Ser927Leu**	27
27	New	c.3187 + 36 C > A	28
28	**New**	**c.3283 G > A p.Glu1095Lys**	29
30	**New**	**c3581G > T, p.Gly1195Val**	7
***MYH7***			
3	Synonym	c.189 C > T, p.Thr63Thr	30, 31, 32
5	**CM034049**	c.502 + 23 T > C	4, 5, 32, 33
		**c.427 C > G; p.Arg143Gly**	3
7	New	c.639 + 31 C > T	30, 31
	Synonym	c.7647 A > G, pAla199Ala	15, 16
	**CM031267**	**c.611 G > A, pArg204His**	34
	**New**	**c.613A > T p.Ser205Cys**	30, 31
	**New**	**c.625 C > G, p.Gln209Glu**	80
8	Synonym	c.732 C > T, pPhe244Phe	35, 36, 37
	**New**	**c. 671 A > T, p.Asn224Ile**	37
9	**CM981329**	**c. 788 T > C, p. Ile263Thr**	18, 27, 38
11	**CM033923**	**C958G > A, p.Val320Met**	39
	Synonym	c.975 C > T, pAsp325Asp	34
12	Synonym	c.1095 G > A, p.Lys365Lys	3
	Synonym	c.1062 C > T, p.Gly354Gly	3
	Synonym	c. 1128 C > T, p.Asp376Asp	37, 40
13	CM930503_rs3218714	c.1208 G > T, p.Arg403Leu	12
	**CM032602**	**c.1231 G > A, p.Val411Ile**	35, 41
	**New**	**c.1252 C > A, p.Gln418Lys**	42
14	**CM003684**	**c.1358 G > A; p.Arg453His**	43
15	New	c.1408-35 T > C	44, 45, 46
	**New**	**c.1486 C > T, p.Leu496Ser**	47
16	**CM920491(G > A)**	**c.1750 G > C, p.Gly584Arg**	33
17	**Harvard**	**c.1954ª > G, p.Arg652Gly**	48
19	**CM054010**	**c.2093 T > C; p.Val698Ala**	39
	**CM941086**	**c.2155 C > T, p.Arg719Trp**	49
20	rs3729818	c.2163-56 A > G	8
	**New**	**c.2189 T > C, p.Ile730Thr**	32
	**CM95212**	**c.2221 G > T, p. Gly741Trp**	50
22	**CM032607**	**c. 2470 G > A, p.Val824Ile**	21
	**Harvard**	**c.2539-2541del; p. Lys847del**	51, 52
	**New**	**c.2585 > T, p.Ala862Val**	13
	**CM952025**	**c.2609 G > A; p.Arg870His**	53
	**CM010347**	**c.2630 T > A; p.Met877Lys**	54
	**CM034055**	**c.2644 C > G; p.Gln882Glu**	43, 45
23	**CM052262**	**C.2702 C > G, p.Ala901Gly**	55
	**CM042418**	**c.2761 G > A; p.Glu921Lys**	56
	**CM920494**	**c.2770 G > A, p.Glu924Lys**	57, 58
24	Synonym	c.267 T > C, p.Ile989Ile	35, 40, 59
32	**CM050712**	**c.4472 C > G, p.Ser1491Cys**	60
37	**CM06819**	**c.5305 C > A, p.Leu1769Met**	47
38	**New**	**c.5573 G > T, p.Arg1858Met**	59
	rs3729833	c.5655 + 32 G > A	3, 35, 59, 61
***TNNT2***			
3	New	c.42-58 G > A	61
4	**CD044989**	**c.53-11_53-7del5 (CTTCT)**	12
7	rs3729843	c.164-50 G > A	62
9	Synonym	c.237 G > A, p.Ser79Ser	63
10	Synonym	c.348 T > C,p.Ile116Ile	64, 65
	**New**	**c.406A > T, p. Arg136Trp**	66
14	**New**	**c.722 A > T, p.Lys241Met**	22
	CM034583_rs3730238	c.779A > G, p.Lys260Arg	67, 68, 69
	**New**	**c.781 + 8 G > A**	33
15	New	c.802-33 C > T	70
	**CM031384**	**c. 833A > T, p.Asn278Ile**	71, 72
16	New	C.843-35 T > C	44, 45, 46
	rs3729998	c.888 + 66 G > A	73
***TNNI3***			
1	**New**	**c.-52 A > T**	22
	rs3729707	c.-35 C > A	22
4	New	c.150 + 27_28CG > GC	4,56, 48
7	**CM031378**	**c.422 G > A, p.Arg141Gln**	45
	**New**	**c.462 G > A, p.Met155Ile**	44, 46
	**CM031379**	**c.470 C > T, p. Ala157Val**	74
	**CM973090**	**c.484 C > T, p.Arg162Trp**	15, 75, 76
	**New**	**c.521A > T, p.Lys174Met**	77
***MYH6***			
2	**New**	**c.220 G > A, p.Gly56Arg**	37
***MYL2***			
3	**New**	**c.132 T > C, p.Ile44Met**	35
***CSRP3***			
3	**CD062135**	**c.128delC, p.Ala43Valfs165**	22, 78, 79
	**New**	**c.170A > C, p.Tyr57Ser**	62
	**CM030827**	**c.131 T > C, p.Leu44Pro**	7

Genetic variants indicated in bold correspond to mutations, others to SNPs

^(a)^Human Genome Mutation Database http://www.hgmd.org and Harvard Sarcomere mutation Database http://genepath.med.harvard.edu/~seidman/cg3/;

**^(b)^**http://www.ensembl.org/Homo_sapiens/Info/Index.

**Figure 1:**
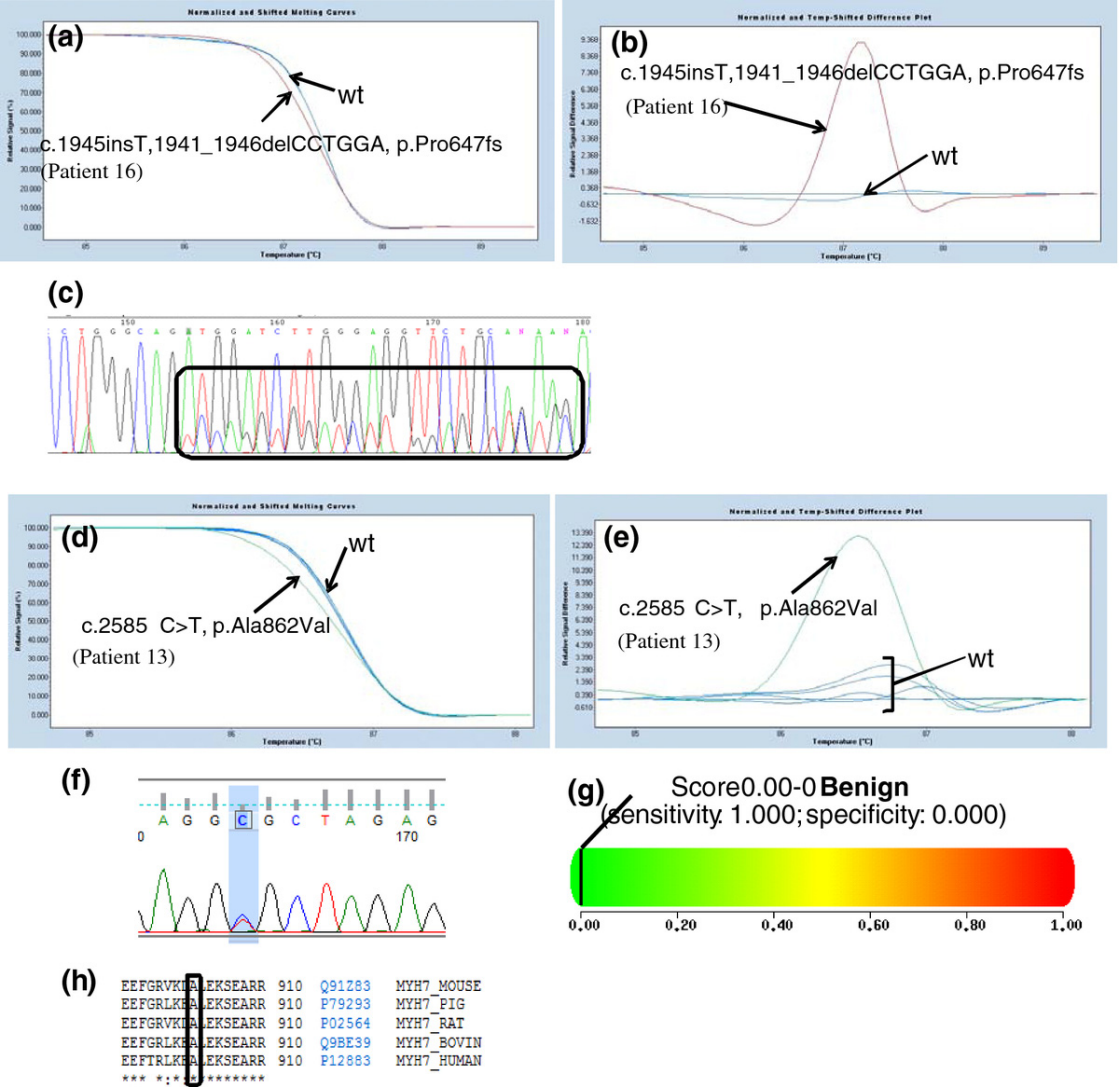
**a) Melting curves of exon 19 of the *MYBPC3 *gene (NM_000256)**. b) Difference plot of the melting curves of exon 19 of the MYBPC3 gene. The arrows in both figures indicate the wild-type (wt) profile and patient 16 respective variations. Two healthy control individuals were used has a reference curve. c) Chromatogram analysis resulting from sequencing exon 19 of the *MYBPC3 *gene in patient 16 with the c.1945insT, 1941-1946delCCTGGA, p.Pro647fs. This mutation was also present in patient 17. d) Melting curves of exon 22(1) of the *MYH7 *gene (NM_000257). e) Difference plot of the melting curves of exon 22(1) of the *MYH7 *gene. The arrows in both figures indicate the wild-type (wt) profile and patient 13 respective variations. Five healthy control individuals were used has a reference curve. f) Chromatogram analysis resulting from sequencing exon 22 of the *MYH7 *gene in patient 13. g) Polyphen analysis of p.Ala862Val genetic variation http://genetics.bwh.harvard.edu/pph2/. h) Conservation across species of codon 862 of the β-myosin protein (source: http://www.uniprot.org/). The box shows the amino acid changed by the *MYH7 *c.2585 C > T mutation. [BOVIN - Bos Taurus; HUMAN - *Homo sapiens*; MOUSE - *Mus musculus*; RAT - *Rattus norvegicus*; PIG - *Sus scrofa*].

**Figure 2:**
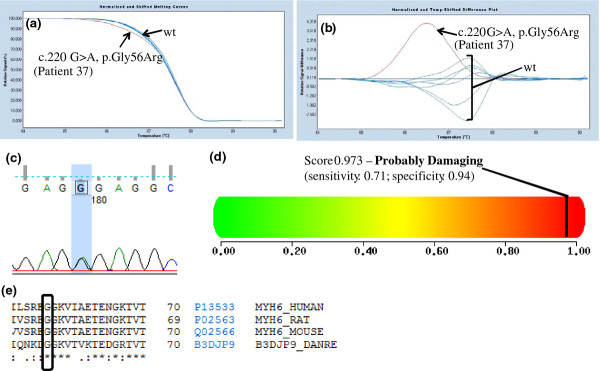
**a) Melting curves of exon 2 of the *MYH6 *gene (NM_002471)**. b) Difference plot of the melting curves. The arrows in both figures indicate the wild-type (wt) profile and the patient respective variation. Seven healthy control individuals were used has a reference curve. c) Chromatogram analysis resulting from sequencing exon 2 of the *MYH6 *gene in patient 37. d) Polyphen analysis of p.Gly56Arg genetic variation http://genetics.bwh.harvard.edu/pph2/. e) Conservation across species of codon 56 of the α-myosin light chain protein (source: http://www.uniprot.org/). The box shows the amino acid changed by the *MYH6 *c.220 G > A mutation. [HUMAN - *Homo sapiens*; MOUSE - *Mus musculus*; RAT - *Rattus norvegicus*; DANRE - *Danio rerio*].

**Figure 3:**
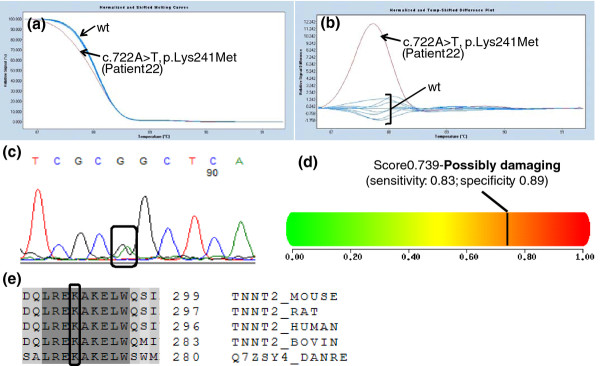
**a) Melting curves of exon 14 of the TNNT2 gene (NM_000364)**. b) Difference plot of the melting curves. The arrows in both figures indicate the wild-type (wt) profile and the patient respective variation. Eight healthy control individuals were used has a reference curve. c) Chromatogram analysis resulting from sequencing exon 14 of the *TNNT2 *gene in patient 22. d) Polyphen analysis of p.Lys241Met genetic variation http://genetics.bwh.harvard.edu/pph2/. e) Conservation across species of codon 241 of the Troponin T protein (source: http://www.uniprot.org/). The box shows the amino acid changed by the *TNNT2 *c.722A > T mutation. [HUMAN - *Homo sapiens; *MOUSE - *Mus musculus; *RAT - *Rattus norvegicus*; BOVIN - *Bos Taurus*; DANRE - *Danio rerio*].

**Figure 4:**
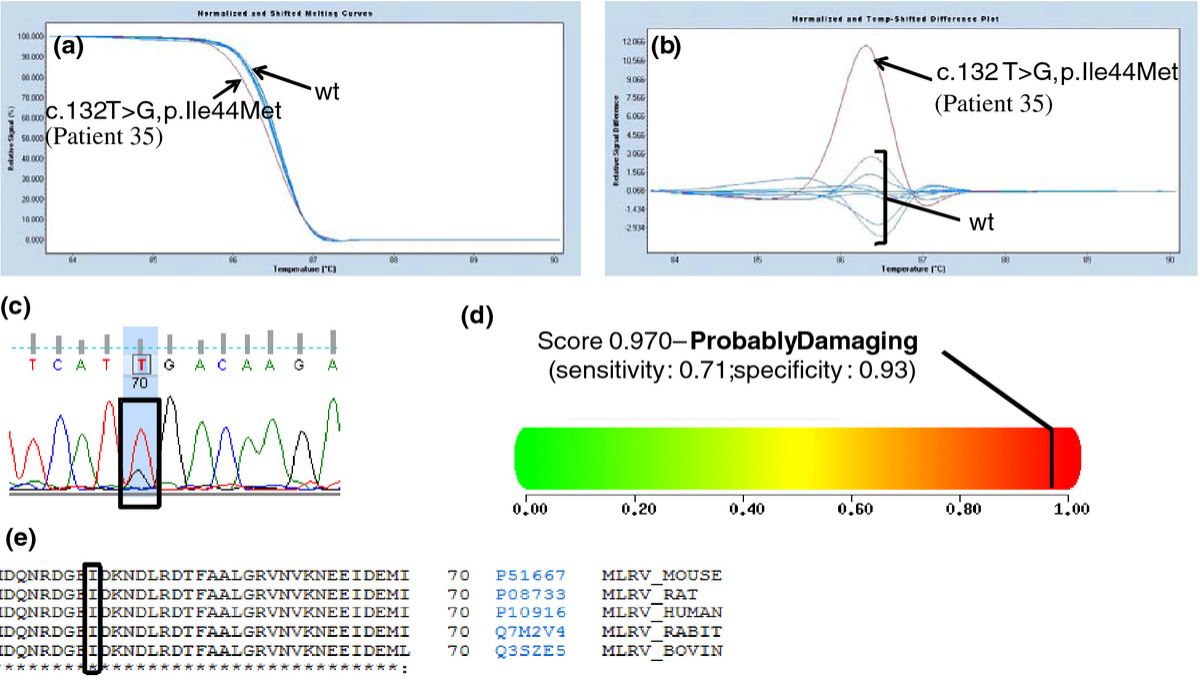
**a) Melting curves of exon 3 of the *MYL2 *gene (NM_000432)**. b) Difference plot of the melting curves. The arrows in both figures indicate the wild-type (wt) profile and the patient respective variation. Eight healthy control individuals were used has a reference curve. c) Chromatogram analysis resulting from sequencing exon 3 of the *MYL2 *gene in patient 35. d) Polyphen analysis of p.Ile44Met genetic variation http://genetics.bwh.harvard.edu/pph2/. e) Conservation across species of codon 44 of the regulatory myosin light chain protein (source: http://www.uniprot.org/). The box shows the amino acid changed by the *MYL2 *c.132 T > G mutation. [HUMAN - Homo sapiens; MOUSE - *Mus musculus*; RAT - *Rattus norvegicus*; BOVIN - *Bos Taurus*; RABIT - *Oryctolagus cunic*].

The novel missense mutations affected highly conserved amino acid residues, and were not found in 200 control chromosomes (Caucasian origin) (Figures 1, 2, 3, 4 and 4 data not shown).

Fourteen patients (17.5%) are carriers of two mutated alleles (patients 3, 7, 10, 11, 13, 21, 27, 33, 35, 37, 39, 43, 45 and 47). Patient 12 is a carrier of three mutated alleles and patient 22 carries three mutated allele plus one 5'UTR mutation with unknown effect (See Additional file 2: Figure S1 and data not shown). In eight patients (10%) no mutated allele was found (patients 8, 19, 36, 40, 63, 64, 65 and 73). This suggests that other genes, not included in this study, or still not yet known may be responsible for the disease in these HCM patients. Nevertheless, compared with previous AS reported detection capacity (no mutation found in 1/3 of the patients) our strategy allowed us an improved characterization of HCM patients [23].

**Figure 5:**
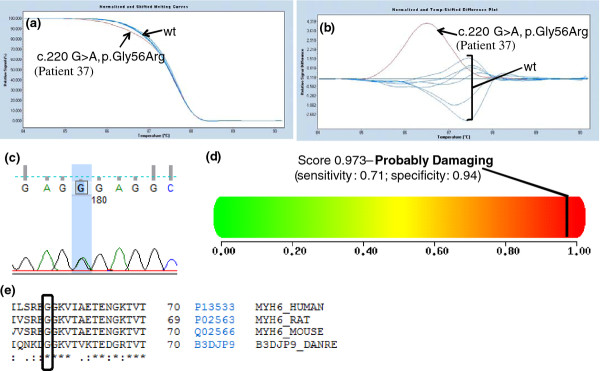
**a) Melting curves of exon 14 of the *TNNT2 *gene (NM_000364)**. b) Difference plot of the melting curves. The arrows in both figures indicate the wild-type (wt) profile and the patients respective variations. Nine healthy control individuals were used has a reference curve. c) Chromatogram analysis resulting from sequencing exon 14 of the *TNNT2 *gene in patient 67. d) Polyphen analysis of p.Lys260Arg genetic variation http://genetics.bwh.harvard.edu/pph2/. e) Conservation across species of codon 260 of the cardiac Troponic T protein (source: http://www.uniprot.org/). The box shows the amino acid changed by the *TNNT2 *c.778A > G mutation. [HUMAN - *Homo sapiens*; MOUSE - *Mus musculus*; RAT - *Rattus norvegicus*; CANFA - Canis familiaris; RABIT - *Oryctolagus cunic*]. f) Melting curves of the population study of exon 14 of the *TNNT2 *gene (NM_000364). g) Difference plot of the melting curves. The arrows in both figures indicate the wild-type (wt) profile and the c.778A > G (p.Lys260Arg) variation on two chromosomes of control individuals. 100 healthy control individuals (200 chromosomes) were used has a reference curve.

In patients 67, 68 and 69 we have found only a single SNP (rs3730238/CM034583) c.779A > G (p.Lys260Arg) in exon 14 of *TNNT2 *gene also described as HCM-associated in the Human Genome Mutation Database http://www.hgmd.org (Figure 5a, b and 5c).

Despite being described as a SNP, and Pholyphen score classify it as benign (Figure 5d), it affects a codon that is conserved in different species (Figure 5e). Moreover, HRM population study of exon 14 of *TNNT2 *gene in 200 chromosomes from healthy control Caucasian Portuguese individuals, allow us to find this allele (rs3730238) in 3 chromosomes from control individuals (1.5%) (Figure 5f and 5g). This SNP has also been found in more European populations, namely in Spanish and French individuals [18,30,31]. Interestingly, it has not been described in more geographically distant populations, such as the Indian population [32]. In this regard, since in these three HCM patients (67, 68 and 69) we were not able to detect other mutated allele, segregation studies within their families are being performed regarding this SNP. We cannot discharge that these 3 patients can have a mutated allele in another gene (not yet known or not analyzed in the current study).

In healthy patients, beside the SNP mentioned above, only synonym changes were found (data not shown). HRM gene scanning data for *MYBPC3 *and *TNNT2 *is presented in Additional file 3: Figure S2 and Additional file 4: Figure S3, respectively.

### *In silico *characterization of novel HCM-causing mutations

The pathogenicity of novel mutations will be further assessed using segregation analysis and two-hybrid based protein functional assays. However, *in silico *studies based on the use of PolyPhen software v2.0.23 http://genetics.bwh.harvard.edu/pph2/ could suggest that most of these novel missense variants are most likely disease causing (Figures 2d, 3d and 4d and results not shown). Two novel mutations in *MYH7 *gene, c.613 A > T (p.Ser205Cys) and c.2585 C > T (p.Ala862Val) in exons 7 and 22, respectively, despite being classified by PolyPhen software as benign, the protein alignments (source: http://www.uniprot.org/) show that the amino acids changed by the mutations are conserved across species (*Homo sapiens; Mus musculus; Rattus norvegicus; Bos Taurus; Danio rerio*) (data not shown and Figure 1g and 1h, respectively).

### Prevalence and spectrum of mutations in the Portuguese population: Correlation with clinical data

Our HRM strategy allowed us to found 36 patients with mutations in *MYH7 *(45%), 24 patients with *MYBPC3 *mutations (30%), 9 patients with *TNNI3 *mutations (11.25%), 6 patients with *TNNT2 *mutations (7.5%), 5 patients with *CSRP3 *mutations (6.25%), 1 patient with a *MYH6 *mutation (1.25%) and 1 patient with a *MYL2 *mutation (1.25%) (Table 3). As previously described by others, in the cohort of HCM patients analyzed in this work, *MYH7 *and *MYBPC3 *mutations accounts for 75% of the HCM cases [this work and [1,7,8]]. Also, this mutation distribution allow us to verify that in our studied HCM cohort there are no prevalent mutations as described in the Netherlands [20] but a marked genetic heterogeneity as most HCM patients have a unique pathogenic mutation such as reported before in the US [1,22]. As reported above, in our cohort we were able to detect 22 novel mutations in *MYBPC3, MYH7, TNNT2, TNNI3, MYH6, MYL2 *and *CSRP3 *genes (Table 3 and Figures 1- 4). Most of these are missense mutations (25%), one is an indel mutation, another a 5' UTR mutation and the two other mutations probably affect splicing of corresponding introns. These novel mutations affect amino acids that are highly conserved among species (Table 3 Figures 1-4 and data not shown) and they were not found in 200 chromosomes from normal Caucasian individuals (data not shown) indicating that they can be the cause of HCM in these patients. Nevertheless, segregation studies are currently being performed in their families.

Moreover, our strategy allowed us to identify mutations in three genes (*MYH6, MYL2 *and *CSRP3)*, not usually analysed by current HCM-genetic diagnostic strategy, in seven HCM patients (8.75%). This fact prompts the importance of a complete gene scanning analysis against the current genetic evaluation (through AS) of the 4 most common HCM-genes (Table 1).

There are several genetic causes for LVH meaning that a differential diagnosis by means of an accurate correlation between clinical data genetic profile could be important considering individualized treatment options and accurate prognosis. All individuals with double mutations (3, 7, 10, 11, 13, 21, 27, 33, 35, 37, 39, 43, 45 and 47) shared a non obstructive septal hypertrophy and had familial history of HCM (Tables 2 and 3). In this regard familial history can be considered as an important parameter to be considered, as individuals 12 and 22 (with three and four mutations, respectively) have familial history of HCM (Tables 2 and 3). Also, mutations in *MYH7 *and *MYBPC3 *genes were involved in all this cases (Table 3). This type of mutational profile correlates to clinical data previously described for HCM-causing mutations in *MYBPC3 *genes [4]. The presence of SNPs in addition to mutations should be considered regarding HCM severity. An example is related to individual 1, which deceased, and has the same mutation as individuals 25 and 26, but with an additional SNP (Tables 2 and 3).

As mentioned before, in order to confirm the co-segregation of the novel mutations with the disease, segregation studies are currently being performed whenever relatives are available for genetic studies and after genetic counseling. In this regard, the mutation c.1945insT1941-1946delCCTGGA, p.Pro647fs was also present in the son of patient 16 (also with clinical diagnostic of HCM) (Figure 4 and 4 results not shown). Nevertheless, patient 16 has a more severe HCM clinical profile (the implantation of a cardiac defibrillator (CDI) was necessary), so most probably individual 16 has another mutation in other DNA region/gene, not analysed under current work (Table 2). Interestingly, patients 44, 45 and 46 co-segregate *MYH7 *and *TNNT2 *mutations within their families (not related), being those intronic variants novel (Table 3 and results not shown). Patient 46 also co-segregates a *TNNI3 *mutation with his father and patient 45 has a mutation in *TNNI3 *gene, not present in both her parents (Table 3 and results not shown). This fact is coincident with HCM genetic profile being characteristic of each family and once again highlights the importance of familial study.

In order to access some more considerations between HCM phenotype and the genetic results presented in this work for our cohort of HCM patients, we observe that apical hypertrophy is mostly related with *MYBPC3 *and *MYH7 *mutations, namely intronic variants, missense and deletions (Tables 2 and 3). Curiously, two patients (69 and 73) with apical hypertrophy presented only a *TNNT2 *genetic alteration (SNP and intronic, respectively), indicating that another DNA region, not yet found, may also be involved in this type of cardiac hypertrophy (Tables 2 and 3). Regarding septal hypertrophy *MYH7*, *MYBPC3*, *MYH6*, *TNNT2 *and *CSRP3 *genes are affected and curiously only one mutation is shared between apical and septal hypertrophy (Tables 2 and 3).

Within our analyzed cohort we had 39 sporadic cases which constitute 48.75% of the HCM cases, being the rest (51.25%) familial cases (Table 2). From the eight patients with no identified HCM-associated mutations six of them (patients 19, 36, 40, 63, 64 and 65) were sporadic cases, being this observation in agreement with previous results [1,7,8]. Nevertheless, all the above considerations regarding genotype and phenotype should be validated using a major sampling of HCM patients with all the necessary clinical characterization/data. Another limitation of our work concerns with the establishment of the effect of novel DNA alterations as the cause of HCM. In order to determine the phenotypic effects of DNA alterations it becomes necessary to perform functional studies, including assessment of protein interaction. In this perspective we initiated functional studies namely of the 5'UTR mutation, the splicing mutations and the indel mutation that are being evaluated by means of two-hybrid system in cardiomyocyte cell lines.

## Conclusions

In our work we intended to improve gene-based diagnostic tools by using a recent high throughput genotyping technology. The rapid, low-cost, and highly efficient HRM strategy fulfills all the conditions required for the systematic detection of genomic variants in the 28 HCM-associated genes, being able to detect DNA variants in 90% of the analyzed HCM patients with 20% of the patients showing more than one mutation (double or compound heterozygosity). We were also able to confirm a marked genetic heterogeneity regarding HCM in our cohort of patients as most patients have a "private" pathogenic mutation. As far as we know this is the first study applying HRM technology for scanning 28 HCM-associated genes in a large cohort of Portuguese Caucasian HCM patients. The elucidation of the molecular basis of HCM cases will provide new insights into genotype/phenotype relationships and will allow a better knowledge of the HCM physiopathology. However, it should be noted that the difficulty in obtaining the clinical data for all our HCM patients, can be considered as a major limitation in order to obtain accurate genotype - phenotype correlations.

## Competing interests

The authors declare that they have no competing interests.

## Authors' contributions

SS, MV, FAR carried out the molecular study and data interpretation analysis. SS, MV, FAR also made substantial contributions to the study conception and design and wrote the paper. PM, SL, OH, LV carried out the molecular analysis. BD, MH, FEJ, FA and GIM identified and diagnosed the patients and obtained the clinical data. CIM and MC participate in the design of the study. All authors read and approved the final manuscript.

## Pre-publication history

The pre-publication history for this paper can be accessed here:

http://www.biomedcentral.com/1471-2350/13/17/prepub

## Supplementary Material

Additional file 1 Table 1**Genomic regions covered in HRM analysis**. Primer sequences and PCR conditions for HCM-associated genes mutation scanning by HRM are described. Click here for file

Additional file 2 Figure 1**a) Melting curves of exon 9 of the *MYH7 *gene (NM_000257)**. b) Difference plot of the melting curves. The arrows in both figures indicate the wild-type (wt) profile and patient 18 respective variations. Three healthy control individuals were used has a reference curve. The altered profile was also obtained for patients 27 and 38.c) Melting curves of exon 11 of the *MYH7 *gene (NM_000257). d) Difference plot of the melting curves. The arrows in both figures indicate the wild-type (wt) profile and patient 39 respective variations. Three healthy control individuals were used has a reference curve. e) Melting curves of exon 14 of the *MYH7 *gene (NM_000257). f) Difference plot of the melting curves. The arrows in both figures indicate the wild-type (wt) profile and patient 43 respective variations. Five healthy control individuals were used has a reference curve. g) Melting curves of exon 19 of the *MYH7 *gene (NM_000257). h) Difference plot of the melting curves. The arrows in both figures indicate the wild-type (wt) profile and patient 39 respective variations. Four healthy control individuals were used has a reference curve. Click here for file

Additional file 3 Figure 2**a) Melting curves of exon 23(1) of the *MYBPC3 *gene (NM_000256)**. b) Difference plot of the melting curves. The arrows in both figures indicate the wild-type (wt) profile and patient 22 respective variations. Two healthy control individuals were used has a reference curve; c) Melting curves of exon 25 of the *MYBPC3 *gene (NM_000256). d) Difference plot of the melting curves. The arrows in both figures indicate the wild-type (wt) profile and patient 7 respective variations. Five healthy control individuals were used has a reference curve. The altered profile was also obtained for patients 25 and 26; e) Melting curves of exon 30 of the *MYBPC3 *gene (NM_000256). f) Difference plot of the melting curves. The arrows in both figures indicate the wild-type (wt) profile and patient 1 respective variations. Four healthy control individuals were used has a reference curve. Click here for file

Additional file 4 Figure 3**a) Melting curves of exon 15 of the *TNNT2 *gene (NM_000364)**. b) Difference plot of the melting curves. The arrows in both figures indicate the wild-type (wt) profile and patient 71 respective variations. Nine healthy control individuals were used has a reference curve. c) Melting curves of exon 7 of the *TNNI3 *gene (NM_000363). d) Difference plot of the melting curves. The arrows in both figures indicate the wild-type (wt) profile and patients respective variations (green curve for patient 74 and red curves for patients 75 and 76). Patient 15 also had a similar red profile. Four healthy control individuals were used has a reference curve. e) Melting curves of exon 3 of the *CSRP3 *gene (NM_003476). f) Difference plot of the melting curves. The arrows in both figures indicate the wild-type profile and patients 22 and 78 respective variations. Ten healthy control individuals were used has a reference curve. The altered profile was also obtained for patient 79. Click here for file
