# Supplementary material for: High resolution melting: improvements in the genetic diagnosis of hypertrophic cardiomyopathy in a Portuguese cohort
Source: BMC Med Genet. 2012 Mar 19;13:17. doi: 10.1186/1471-2350-13-17 (PMC3359199; doi:10.1186/1471-2350-13-17)
Supplement: Additional file 1 — Table 1Genomic regions covered in HRM analysis. Primer sequences and PCR conditions for HCM-associated genes mutation scanning by HRM are described. [file 1471-2350-13-17-S1.PPT]

## Slide 1
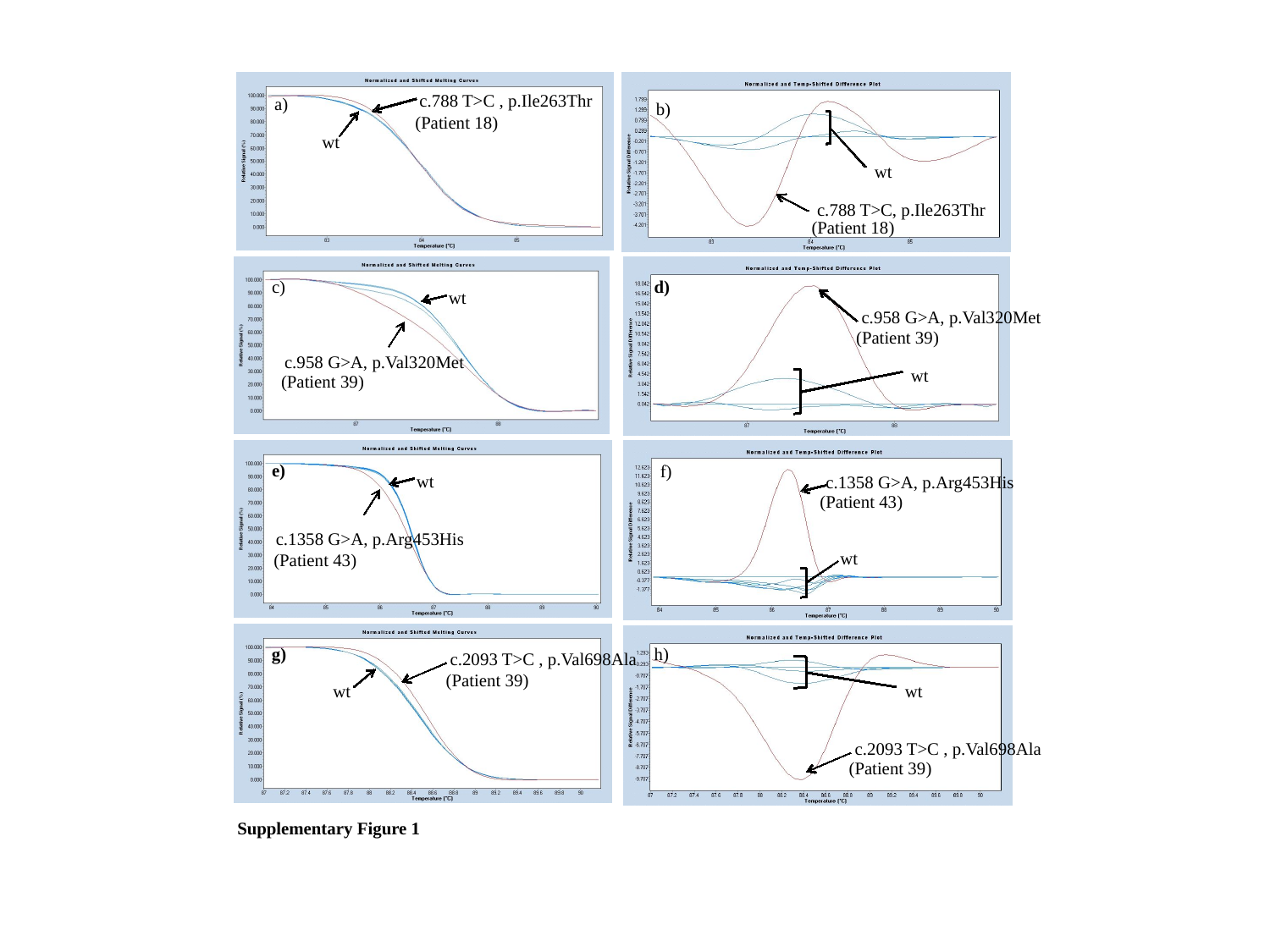

c.788 T>C , p.Ile263Thr
a)
b)
wt
wt
c.788 T>C, p.Ile263Thr
c)
d)
wt
c.958 G>A, p.Val320Met
c.958 G>A, p.Val320Met
wt
e)
f)
wt
c.1358 G>A, p.Arg453His
c.1358 G>A, p.Arg453His
wt
g)
h)
c.2093 T>C , p.Val698Ala
wt
wt
c.2093 T>C , p.Val698Ala
(Patient 18)
(Patient 18)
(Patient 39)
(Patient 39)
(Patient 43)
(Patient 43)
(Patient 39)
(Patient 39)
Supplementary Figure 1
