# Supplementary material for: High resolution melting: improvements in the genetic diagnosis of hypertrophic cardiomyopathy in a Portuguese cohort
Source: BMC Med Genet. 2012 Mar 19;13:17. doi: 10.1186/1471-2350-13-17 (PMC3359199; doi:10.1186/1471-2350-13-17)
Supplement: Additional file 2 — Figure 1a) Melting curves of exon 9 of the MYH7 gene (NM_000257). b) Difference plot of the melting curves. The arrows in both figures indicate the wild-type (wt) profile and patient 18 respective variations. Three healthy control individuals were used has a reference curve. The altered profile was also obtained for patients 27 and 38.c) Melting curves of exon 11 of the MYH7 gene (NM_000257). d) Difference plot of the melting curves. The arrows in both figures indicate the wild-type (wt) profile and patient 39 respective variations. Three healthy control individuals were used has a reference curve. e) Melting curves of exon 14 of the MYH7 gene (NM_000257). f) Difference plot of the melting curves. The arrows in both figures indicate the wild-type (wt) profile and patient 43 respective variations. Five healthy control individuals were used has a reference curve. g) Melting curves of exon 19 of the MYH7 gene (NM_000257). h) Difference plot of the melting curves. The arrows in both figures indicate the wild-type (wt) profile and patient 39 respective variations. Four healthy control individuals were used has a reference curve. [file 1471-2350-13-17-S2.PPT]

## Slide 1
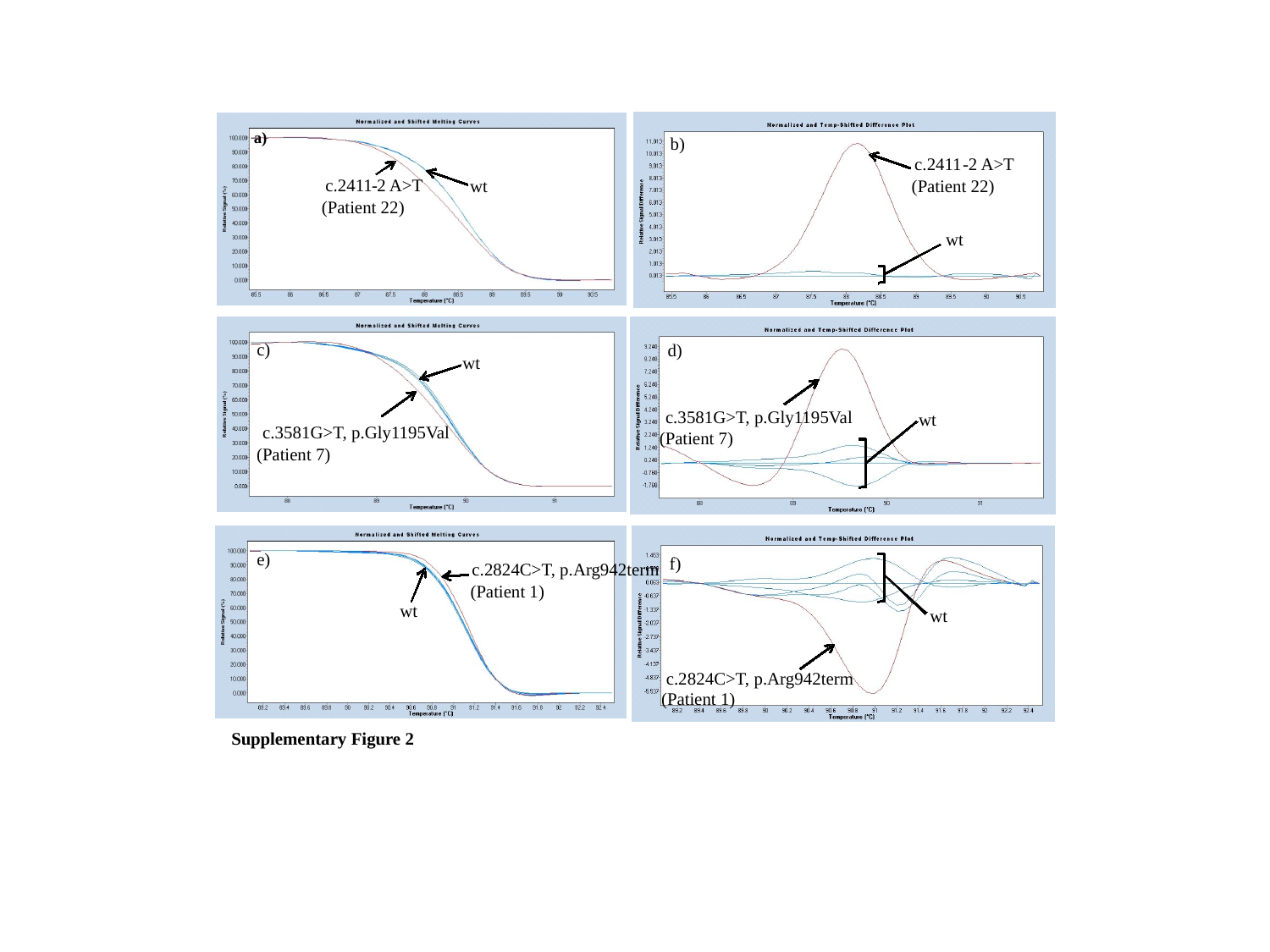

a)
b)
c.2411
-2 A>T
c.2411
-
2 A>T
wt
(Patient 22)
wt
c)
d)
wt
c.3581G>T, p.Gly1195Val
wt
c.3581G>T, p.Gly1195Val
e)
f)
c.2824C>T, p.Arg942term
wt
wt
c.2824C>T, p.Arg942term
(Patient 22)
(Patient 7)
(Patient 7)
(Patient 1)
(Patient 1)
Supplementary Figure 2
