# Supplementary figures and images for: High resolution melting: improvements in the genetic diagnosis of hypertrophic cardiomyopathy in a Portuguese cohort
Source: BMC Med Genet. 2012 Mar 19;13:17. doi: 10.1186/1471-2350-13-17 (PMC3359199; doi:10.1186/1471-2350-13-17)

## Slide 1
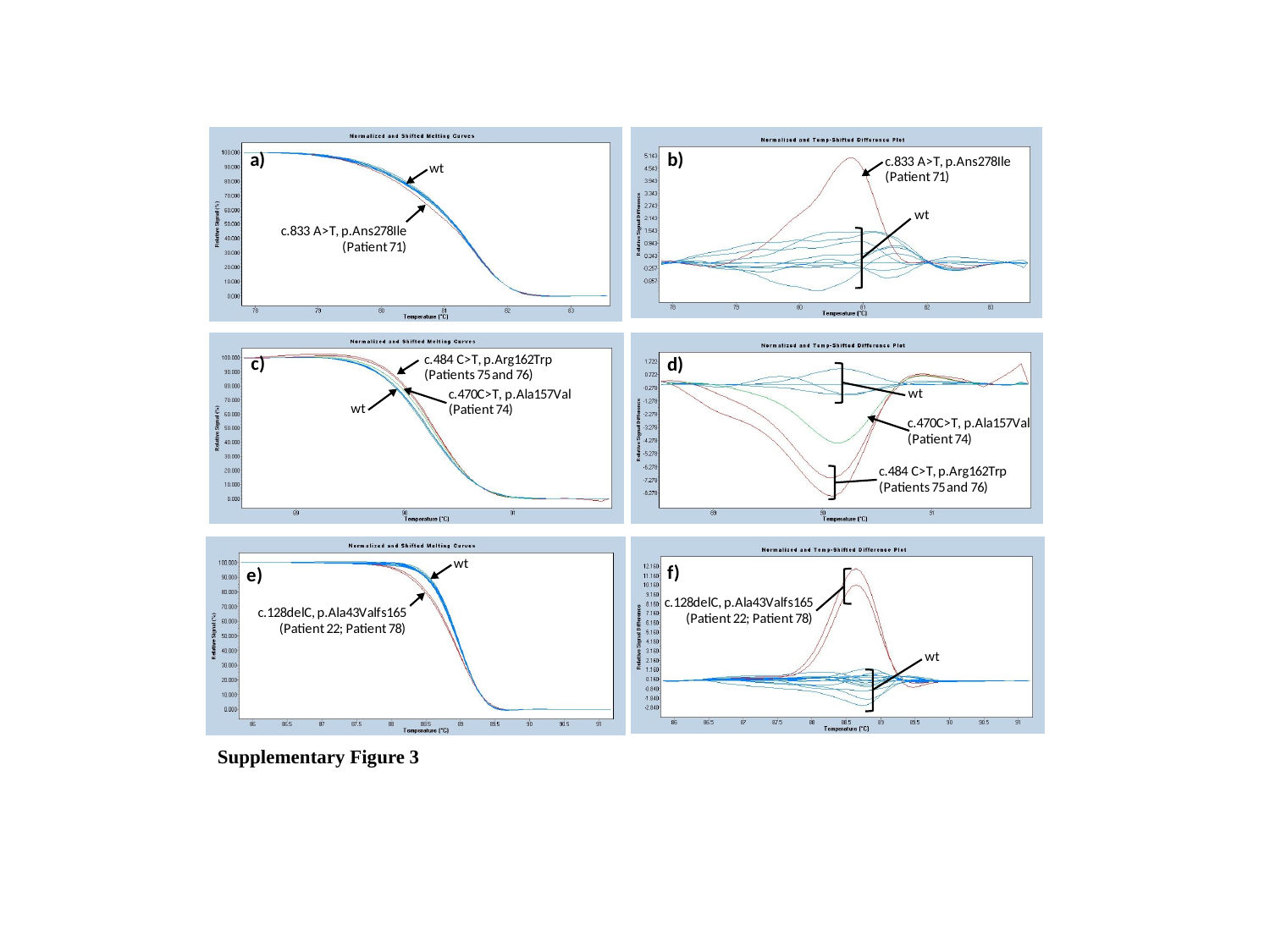

Supplementary Figure 3

Supplement: Additional file 3 — Figure 2a) Melting curves of exon 23(1) of the MYBPC3 gene (NM_000256). b) Difference plot of the melting curves. The arrows in both figures indicate the wild-type (wt) profile and patient 22 respective variations. Two healthy control individuals were used has a reference curve; c) Melting curves of exon 25 of the MYBPC3 gene (NM_000256). d) Difference plot of the melting curves. The arrows in both figures indicate the wild-type (wt) profile and patient 7 respective variations. Five healthy control individuals were used has a reference curve. The altered profile was also obtained for patients 25 and 26; e) Melting curves of exon 30 of the MYBPC3 gene (NM_000256). f) Difference plot of the melting curves. The arrows in both figures indicate the wild-type (wt) profile and patient 1 respective variations. Four healthy control individuals were used has a reference curve. [file 1471-2350-13-17-S3.PPT]
