# Supplementary material for: High resolution melting: improvements in the genetic diagnosis of hypertrophic cardiomyopathy in a Portuguese cohort
Source: BMC Med Genet. 2012 Mar 19;13:17. doi: 10.1186/1471-2350-13-17 (PMC3359199; doi:10.1186/1471-2350-13-17)
Supplement: Additional file 4 — Figure 3a) Melting curves of exon 15 of the TNNT2 gene (NM_000364). b) Difference plot of the melting curves. The arrows in both figures indicate the wild-type (wt) profile and patient 71 respective variations. Nine healthy control individuals were used has a reference curve. c) Melting curves of exon 7 of the TNNI3 gene (NM_000363). d) Difference plot of the melting curves. The arrows in both figures indicate the wild-type (wt) profile and patients respective variations (green curve for patient 74 and red curves for patients 75 and 76). Patient 15 also had a similar red profile. Four healthy control individuals were used has a reference curve. e) Melting curves of exon 3 of the CSRP3 gene (NM_003476). f) Difference plot of the melting curves. The arrows in both figures indicate the wild-type profile and patients 22 and 78 respective variations. Ten healthy control individuals were used has a reference curve. The altered profile was also obtained for patient 79. [file 1471-2350-13-17-S4.DOC]

**Supplemmentary Table I –** Genomic regions covered in HRM analysis. Primer sequences and PCR conditions for HCM-associated genes mutation scanning by HRM are described.

| **Gene** | **Exão** | **Forward primer (5’ 🡪 3’)** | **Reverse primer (5’ 🡪 3’)** | **Amplicon**  **(bp)** | **[Primer]**  **(µM)** | **[MgCl_2_] (mM)** | **Ta (ºC)** |
| --- | --- | --- | --- | --- | --- | --- | --- |
| ***ACTC1*** | 2 | CAGGCAGCTAAGCGTGGT | ACTGAAGGGGTCCCGAGT | 240 | 0,5 | 2,5 | 55 |
|  | 3(1) | TTCCTGACATGGTGAGAGCA | CTTCTCCATGTCGTCCCAGT | 176 | 0,5 | 2,5 | 57 |
|  | 3(2) | ACTGGGACGACATGGAGAAG | TTCAACTGGGGGATCTGATT | 299 | 0,5 | 2,5 | 56 |
|  | 4 | TGGCTAGAGCAGTGGTGTTG | GGTAGGCGGATTCAGTGAGA | 293 | 0,5 | 2,5 | 57 |
|  | 5(1) | TCACTGAATCCGCCTACCTC | AGCTGTGGCCATCTCATTCT | 155 | 0,5 | 2,5 | 56 |
|  | 5(2) | GAACGTGAAATTGTCCGTGA | AGGGAAAATCGTGCCTCTG | 229 | 0,3 | 2 | 55 |
|  | 6 | ACCTTGACCTGAATGCACTGT | TCGGATCTCCCACTCACAA | 293 | 0,5 | 2,5 | 56 |
|  | 7 | TGGAGTCTTCCAAACCACCT | CCTACCCCAAAAACAAACGA | 159 | 0,5 | 2,5 | 55 |
| ***CASQ2**** | 1 | ACGTTGGATGTTGCCTCTACTACCATGAGC | ACGTTGGATGGCCCTTTGGTTACTTACCTC | 116 | 0,5 | 2,5 | 55 |
| ***CAV3**** | 2 | ACGTTGGATGGCCCTTTGGTTACTTACCTC | ACGTTGGATGTGTGGGCACCTACAGCTTTG | 95 | 0,5 | 2,5 | 55 |
| ***CSRP3*** | 1 | CTCATTCACCCTCTCCCTTG | AAACCAGCCACAGAACCAAC | 190 | 0,5 | 2,5 | 55 |
|  | 2 | GGGGCCACTTCAATGTAGG | TGTGATGCTGTCCGGATG | 233 | 0,5 | 2,5 | 55 |
|  | 3 | TCAATTCCAATCCCATGACC | TGGAGACTTTAACAGGCAAGG | 248 | 0,5 | 2,5 | 55 |
|  | 4 | TTTGCCAAGGGAAATCTACG | CCTGGTGGAGATGAGCAAAT | 228 | 0,5 | 2,5 | 55 |
|  | 5 | ACAGAATGTGTTGCCTACCTCA | AGGGCCCTTTTAGGGAAAAC | 197 | 0,5 | 2,5 | 55 |
|  | 6(1) | CGTGCCTGGCGAACTTAT | TGGGGAAAGGTATCGATCTG | 296 | 0,5 | 2,5 | 55 |
|  | 6(2) | TGCACAGATCGATACCTTTCC | TTATATGCTCTGCAACTCGTTTTT | 287 | 0,3 | 3 | 56 |
|  | 6(3) | TGGAATGGGAGAGGCAATAA | TTTTGATTTTCCTTTTAGATTTTGCT | 300 | 0,3 | 2,5 | 56 |
|  | 6(4) | GGGGGTCTGGGAGAAAATAG | GCTAAACCCCAGGGAATTGT | 225 | 0,5 | 2,5 | 55 |
| ***COX15*** | 5 | CCTTGTTTTGTTTGCTTCATCTC | GCGGGGTCTTGAACACAG | 243 | 0,5 | 2,5 | 56 |
| ***DES*** | 9(1) | ATGGTTGGACTGGGCTTCT | ATGGGCTATGTCGCTGTTG | 244 | 0,5 | 2,5 | 55 |
|  | 9(2) | GTCCCCAACAGCGACATAG | AGTCCTGGCCCTGCAGTAT | 237 | 0,5 | 2,5 | 56 |
|  | 9(3) | GATACTGCAGGGCCAGGAC | CATCTCACCCACTTTCTCTCCT | 249 | 0,5 | 2,5 | 58 |
| ***FXN*** | 1 | ACAGCTAGGAAGTGGGCAGA | ACGGAGTGCAACCAGGAC | 227 | 0,5 | 2,5 | 55 |
| ***JPH2*** | 1(1) | AGTGGTGATGGGGCTGAG | GGGAAGGAGGGGTATTTCAA | 290 | 0,5 | 3 | 55 |
|  | 1(2) | CTGCCTGCACTCAGTTCTCA | CCCCCTTCTCCAATCCTG | 214 | 0,5 | 2,5 | 56 |
|  | 1(3) | CAGGGGACTCTGAGAATGGA | CTGGGACGGAAAGGTCAGT | 253 | 0,5 | 2,5 | 55 |
|  | 2(1) | CTACTGACGCGCCCTCTC | ATTGGCCAGGAGGCTGAG | 271 | 0,5 | 2,5 | 55 |
|  | 2(2) | TCGCGCTCAGCCTCCT | GTTTGTCGTTCTTCCACTCG | 300 | 0,5 | 2,5 | 55 |
|  | 2(3) | CTACATGGGCGAGTGGAAGA | CTGGAGGCGGCAATCTC | 300 | 0,5 | 2,5 | 55 |
|  | 3 | GGCTGGTTCTCTGGAAGGAT | AGAAAAGCGCCCACCCTA | 229 | 0,5 | 2,5 | 55 |
|  | 4(1) | GGATCACCCAACAGTGGTG | CAGGCCGTCCTTGGACAC | 287 | 0,5 | 2,5 | 55 |
|  | 4(2) | CTGCACGAGCGTGAGACC | GCTGTGGTAGCCCTGGTAAA | 291 | 0,5 | 2 | 56 |
|  | 4(3) | GCTTTACCAGGGCTACCACA | CTTCCGCGCCTTCTTCTT | 286 | 0,5 | 2,5 | 55 |
|  | 5 | CTCTGTCCAGCCTTGCTGT | CACCCTGGTGCTCAAAACTC | 246 | 0,5 | 2,5 | 55 |
| ***LAMP2**** | 6 | ACGTTGGATGTTAGACTCAATAGCAGCACC | ACGTTGGATGGTGTTTCTAAGAGAATGAACC | 116 | 0,5 | 2 | 55 |
| ***MYBPC3*** | 1 | ACCCCACTCAGTCCCTCTTT | GCTCCCCAATTGTAGACACC | 152 | 0,5 | 2,5 | 57 |
|  | 2(1) | TGCTAGCACAGTATTTACTGAGAGG | CCGCACTGTCAGCGTATG | 270 | 0,5 | 2,5 | 56 |
|  | 2(2) | CAGCGCCAGCAACAAGTA | GTGAAAGCACCTCCTGTTCC | 242 | 0,3 | 2,5 | 55 |
|  | 3 | GCGGGAGGACAGCCATGGCAGAC | CGCCCTACCCACGGATCCTGCC | 286 | 0,5 | 2,5 | 56 |
|  | 4 | GGCTGAGGCAGGAGAATGGTGT | AGGGGCGACAGGCCCGGCTT | 312 | 0,5 | 2 | 58 |
|  | 5 | CAGCAGGACACTCCCCAAG | GTCCCCTCTCTCCGTGTCTC | 235 | 0,3 | 2,5 | 55 |
|  | 6 | GGCCACTCCCAGTCTCCT | AGCCCAGGACAGACACCA | 234 | 0,5 | 2 | 55 |
|  | 7* | ACGTTGGATGCATCTCTCCACCCTTTGAAC | ACGTTGGATGTCAGACTCCAGCACTGGCCT | 141 | 0,5 | 2,5 | 57 |
|  | 8 | CCTTCAGGGTCTCGACTGG | CTGCGGATGGTGCAGGTAG | 192 | 0,5 | 2,5 | 55 |
|  | 9 | CCACTCCACTCCCATCCTGC | ACAGTGCTGGGATTTGGAGCC | 192 | 0,5 | 2,5 | 55 |
|  | 10 | GCGGAAAATAGGGAGGAAGT | GCTCCTGGCAGAATTAGGG | 185 | 0,5 | 2,5 | 55 |
|  | 11(1) | GTGGCTACAGCTCCTTGGTC | CCTGTGCTCTTCTTCTCATCG | 256 | 0,5 | 2,5 | 58 |
|  | 11(2) | CAGTACGGCGTCACTGACC | CCTGTGTAGGGAAGGGCTAG | 134 | 0,5 | 2,5 | 58 |
|  | 12 | TAGCTTGGCCTGGGGGAGCA | AGAGATACGCATGTGGAGAGG | 284 | 0,5 | 2,5 | 55 |
|  | 13 | CAGCTTTCCTGCCACTTCC | CAGGTTCCCACATCCTCAG | 193 | 0,5 | 2,5 | 55 |
|  | 14 | CCAACCCTCATGCTCACC | CAAGTGCTGTGGCCTCTTCT | 201 | 0,5 | 2,5 | 55 |
|  | 15 | GGCATCTGCCCAGAAGAG | AGGGGTCCAAGCCCTAAAG | 265 | 0,5 | 2,5 | 55 |
|  | 16 | CGAGCTCAATGGCTCTGC | CATCTCAGTCTCCACCTGTCC | 241 | 0,5 | 2,5 | 57 |
|  | 17* | ACGTTGGATGCTGCAGGGTCCACAAACTGA | ACGTTGGATGAGCAGGCTCACCCATGAAGT | 144 | 0,3 | 2,5 | 60 |
|  | 18* | ACGTTGGATGGTGCTACTTGCTCTTCCTTC | ACGTTGGATGTGAGCAGAACCAAGACTCAG | 114 | 0,2 | 2 | 56 |
|  | 19* | ACGTTGGATGCTCCCGTTTCTCTGAACTAC | ACGTTGGATGACTTGGCTGGTTCCACACAC | 257 | 0,3 | 2,5 | 60 |
|  | 20* | ACGTTGGATGTACTTCCCTCCTGCCCTGTT | ACGTTGGATGTGAAAGACAAACGAGCCTCC | 168 | 0,5 | 2,5 | 55 |
|  | 21* | ACGTTGGATGATCTCACCCCAACTCTGCAC | ACGTTGGATGCCTCTGTGTTCTCCAGCTTG | 226 | 0,5 | 2,5 | 55 |
|  | 22 | GCTGATGTGGGTCCATCC | GAGGTGGCAGCTCTGGTCT | 234 | 0,5 | 2 | 58 |
|  | 23(1) | CCTGGGTTCCAGACCAGAG | CCTGGGTTCCAGACCAGAG | 168 | 0,5 | 3 | 56 |
|  | 23(2) | CGGCTGAACTTCGACCTGATT | CCTGCAGAGCACCTGCTATTA | 221 | 0,5 | 2 | 58 |
|  | 24 | GATCTCCAGCTTCCCCAGGC | GGTGTCCTCAACTTTCGGCAA | 310 | 0,5 | 2,5 | 55 |
|  | 25* | ACGTTGGATGTCAGTGGTGACACAGCCTG | ACGTTGGATGTCTTGTGACTGCACAAAGGG | 264 | 0,5 | 2,5 | 58 |
|  | 26* | ACGTTGGATGCCCTCACTTAGCTACCCACT | ACGTTGGATGACACACTATAGCCTCTCTCC | 193 | 0,5 | 2,5 | 55 |
|  | 27 | CCCTTGGAGTGATCCAGGT | CCAGGGAAGGGAAACAAGG | 288 | 0,5 | 2,5 | 56 |
|  | 28* | ACGTTGGATGTCACTGTCAGGAGGCGTGGT | ACGTTGGATGTGAAGGGTAGCTGCGGCCTG | 279 | 0,5 | 2,5 | 55 |
|  | 29 | AGAGGCTCTCGGCATCAG | CCTCTCCCTGTTCCCACA | 286 | 0,5 | 2 | 58 |
|  | 30* | ACGTTGGATGTAGCTTTGTGTGGCCCTCTC | ACGTTGGATGCTGGACCAGCGCCTAAAGT | 227 | 0,2 | 2 | 55 |
|  | 31 | GCTTTGCTCCGTTGTCCTC | AGCCTCCCATTTACTGATGG | 250 | 0,5 | 2,5 | 55 |
|  | 32 | AAGGCTGGGAGGACACAGT | GACTTGTGCCCTGGGTGT | 110 | 0,3 | 2 | 56 |
|  | 33(1) | GCCCTCCATTCACTCGTAAG | CCCCAGACATTGTTTCTTGAG | 271 | 0,5 | 2,5 | 55 |
|  | 33(2) | TTGCACAGACTGGTCCACAG | GAAAACAGGCACACCGAAAT | 193 | 0,5 | 2,5 | 55 |
|  | Int 8* | ACGTTGGATGTGGACCTCCTATCAGCCTTC | ACGTTGGATGTCAGACTCCAGCACTGGCCT | 96 | 0,5 | 2,5 | 55 |
|  | Int 15* | ACGTTGGATGAGCCAACCCTCATGCTCAC | ACGTTGGATGATCACCAGCTGGTCCTCCAA | 98 | 0,5 | 2,5 | 55 |
|  | Int 23* | ACGTTGGATGTGCGTCTGGCACGTCTGGAT | ACGTTGGATGACGAGCAACGTTACTCAAGG | 98 | 0,5 | 2,5 | 55 |
| ***MYH6*** | 2 | TGCGTCTTTCCCTTTCTGAC | GAAGCATGCCCCAGTCTCT | 240 | 0,3 | 3 | 57 |
|  | 3 | CAGGAGTAACATAGCCCTCCTGT | TCTTTCCCAGACCTCCTTCC | 297 | 0,3 | 2,5 | 55 |
|  | 4 | GGTCACTCATCCTCCTGCTT | TGGCTTATTTAGGCCTCCAC | 240 | 0,5 | 2 | 56 |
|  | 5 | CTCATGCCCAGCCTTGTC | GGAGGAGGAGCAGAGACCA | 240 | 0,5 | 2 | 55 |
|  | 6 | ATGCTGAGCCCTGTATGGAG | TTAGGGGTAACTCGGGTCAG | 240 | 0,5 | 2 | 56 |
|  | 7 | ATGCTGAGCCCTGTATGGAG | TTAGGGGTAACTCGGGTCAG | 243 | 0,5 | 2 | 57 |
|  | 8 | TCCCTCACTCTGTCCCATTC | AGGCGAGAAGATGTGGCTTA | 243 | 0,5 | 2 | 56 |
|  | 10 | TCCTTCCTCACCTGCCTTC | AGTCGTTGGGGTGTGCAG | 195 | 0,5 | 2,5 | 56 |
|  | 11+12 | ATGGCCACCGATGTGAGT | GCCTGGTCAGCACCTCAG | 296 | 0,5 | 2,5 | 56 |
|  | 13 | GAAGTGCTCACTTATCCTTTCC | GCGATGTCCAGGACTCCTAT | 283 | 0,5 | 2 | 56 |
|  | 20 | CACCCTGGATACTCCCCTCT | TCTAGTGCATGCCTCCCTTT | 228 | 0,3 | 3,5 | 56 |
| ***MYH7*** | 1 | CCCATATATACAGCCCCTGAGA | GCACATACGCCCATGTTTAG | 235 | 0,5 | 3 | 56 |
|  | 2 | AGAAGCAGGAAGGTGGGACT | CCAGCACATGGCAGAAACTA | 224 | 0,5 | 2,5 | 56 |
|  | 3* | ACGTTGGATGTTCTGCTCACTCCAGGCACA | ACGTTGGATGACACCCACCTTGCCATACTC | 252 | 0,5 | 2,5 | 55 |
|  | 4 | TGAGCACTATTGCCCTGTCA | CATGGATGGAGCAAGAACAG | 294 | 0,3 | 2,5 | 55 |
|  | 5* | ACGTTGGATGCACTGCTCCTTTTCTATCCC | ACGTTGGATGAGTTCCCTTCAGGAAGACCC | 236 | 0,2 | 2 | 56 |
|  | 6 | GAGGGAGAAGGAAGGGAGAA | GCTGGGATCAGGGAGATTC | 156 | 0,5 | 2 | 56 |
|  | 7 | CCAGGCATTCTCTCCTGATT | TCTTCTCCCTCCCTTTCTGC | 239 | 0,3 | 2,5 | 55 |
|  | 8 | GCTCTCACCTGCCTCCTTC | TTCCTCCACCAGTCCAAGTC | 172 | 0,2 | 2,5 | 56 |
|  | 9* | ACGTTGGATGACTCATCACCACTCTCTTCC | ACGTTGGATGTAGAGCAAGGGTGAGCTTAG | 163 | 0,2 | 2 | 56 |
|  | 10 | TCTGCCTTTTGCTTGCTACA | ACCAGGTTGCCATGGAGATA | 240 | 0,3 | 2,5 | 55 |
|  | 11 | CTCAGGCCATGTGCTGTGG | TGCCAATCCTCCCACCCC | 226 | 0,5 | 2,5 | 56 |
|  | 12 | GGGATCTCACTTACCCATCATACT | CCCTCCATGACTTGACAGC | 249 | 0,3 | 2,5 | 55 |
|  | 13 | CTGGCCAGCAGTCATCTCTT | CTGCCCACCCATTATCATCT | 205 | 0,3 | 2,5 | 56 |
|  | 14 | CCCTGCTCAATATGGGTCTC | GGTCCACAGCTGGCTCTAAG | 271 | 0,2 | 2,5 | 55 |
|  | 15 | GCCACTCACACCCACTTTCT | GCACACCCAACAAGAACACCA | 276 | 0,5 | 2 | 58 |
|  | 16(1) | GAGCAGAATCCATGTCACCT | TGCAGCCAGCCAATGATGTT | 268 | 0,5 | 2,5 | 56 |
|  | 16(2)* | ACGTTGGATGAAGCCTGAAGCCCACTTCTC | ACGTTGGATGTCCCTACTTACGCGCATCAG | 206 | 0,5 | 2,5 | 55 |
|  | 17 | CCTACCTCCCCACACTGATG | GGGAGGAGTAGGGGATGAAC | 211 | 0,5 | 2 | 58 |
|  | 18 | TGCATCTCTTTCTGGCATTTT | TGTCCTAGGAGGTCCTGTTCC | 212 | 0,3 | 2,5 | 55 |
|  | 19* | ACGTTGGATGCTACTTCCTTCTTGCCACAG | ACGTTGGATGCTGTTCTATGAGCTCTGGTG | 193 | 0,2 | 2 | 56 |
|  | 20 | CAGATCACTGCAGAGCATGG | CAACAGGAAAAGCATCAGAGG | 243 | 0,3 | 2,5 | 55 |
|  | 21 | CCTCGTACCCCTCCCTAGTC | CTCAGAGAAGCGGGAAACCT | 221 | 0,2 | 2,5 | 58 |
|  | 22(1) | AGGCTCAGCACTCCTTTCAA | TCTTCTCCTCCAGCTCCTTG | 239 | 0,5 | 2,5 | 55 |
|  | 22(2) | CCTCAAAGAGGCGCTAGAGA | GGGTGGAAGAGCCAACAGTA | 141 | 0,5 | 4 | 55 |
|  | 23 | AACAGCCTCCCCTCTGTTC | CATCGATGTCCCTTTTGAGC | 217 | 0,5 | 2,5 | 55 |
|  | 24 | GCTGGTGACCTTTGACCCTA | GGCCCCACAACTCTCAATC | 249 | 0,5 | 2,5 | 55 |
|  | 25 | TCCTGAGGTAACTGAACAACAAAAT | TTGGGTCTGCTTGTACTGTTATG | 287 | 0,5 | 2,5 | 55 |
|  | 26 | CCCACGAGTCTCCCTTACCT | GCAGGGGAAACAGAACCAG | 192 | 0,5 | 2,5 | 55 |
|  | 27(1) | GCTAAACTGACTTGCTGTTCCA | CTCGCGCTTCTTGTTCATCT | 273 | 0,5 | 3 | 56 |
|  | 27(2) | GCCACGTCCGTGCAGATCGA | GAGGAAGTTGGAGGAGGGGA | 298 | 0,5 | 2,5 | 56 |
|  | 28 | GCACCTCTTACACCCCTTCA | TCAGGAGGTTGGGGAGACT | 250 | 0,5 | 3 | 55 |
|  | 29 | AGAGGAGGAGGTGGGGATAG | TGCAAGGCTAGTCAGTGTGC | 231 | 0,5 | 2,5 | 58 |
|  | 30(1) | AAGGGTGGGGTTGCTTTAT | TTGGCCTCCTCGAGCTCCTCA | 258 | 0,5 | 2,5 | 55 |
|  | 30(2) | CCAGTGGAGGACCAAGTATGA | CCCTGAGAGGAGAAGGAGGT | 219 | 0,3 | 2 | 55 |
|  | 31 | ATCCACACCCTCCATCCTC | CCTCTCACTGAACCCCTCAT | 298 | 0,5 | 2,5 | 55 |
|  | 32 | GGGGGCTGAAGAGTGAGC | CCTGCAGGTTTTTGTTCTCC | 199 | 0,5 | 3 | 55 |
|  | 33 | GCGTCTGGGCAAAGAGGCGTGTC | TCATCCCCCGCTTTGTCCGTTTTG | 292 | 0,5 | 3 | 55 |
|  | 34 | CTGTGCCCTGACTGTCTGC | AGCTGGATCTCCATCTCATTG | 290 | 0,5 | 2 | 55 |
|  | 35 | CCTGCTCATGCCCACTCT | AGCCTGTGCTCCCTTCAG | 275 | 0,5 | 2,5 | 57 |
|  | 36 | CTGGGGCTCAGCAAGCAA | AGCCCACGGAGAGACACT | 261 | 0,5 | 2,5 | 57 |
|  | 37(1) | AAGTGTGTGAGGACTTGACCAG | AGCTTCTGCAGCTGCTTCTT | 212 | 0,5 | 2,5 | 55 |
|  | 37(2) | GAGCAGATCGCCCTCAAG | CTCTTCATTCTCCTCAGCTGGT | 234 | 0,5 | 2,5 | 55 |
|  | 38 | CCTTCTATGACTGTGCCATCTTC | TTCTCAGACTCCTGGCTTGG | 220 | 0,5 | 2,5 | 57 |
|  | 39 | CCCCTCTCACCTCATGCTC | TCTGTCTGGGTATGCCTGCT | 224 | 0,5 | 2,5 | 56 |
|  | 40 | GCCCAATACCATCTCTCCAA | CACTCCCCTGCATTTCCA | 267 | 0,5 | 2,5 | 55 |
| ***MYL2*** | 1 | ACTTCAGAAGAACGGCATGG | CGCTTGTAGTGGCTTCCTCT | 250 | 0,5 | 2,5 | 56 |
|  | 2* | ACGTTGGATGAGGCACCTAAGAAAGCAAAG | ACGTTGGATGCCCACCTCCTTAAATTCCTG | 117 | 0,5 | 2,5 | 59 |
|  | 3 | AATCTCTTGGGCAACTCTGC | CTCCTGCTCCTCATGGATGT | 202 | 0,5 | 2,5 | 55 |
|  | 4 | GGAATCCCAGGAGCCAAT | CCCCCGAAGAAACATAGACA | 244 | 0,5 | 2,5 | 56 |
|  | 5 | CCTTCATCTCTGGGGGAACT | TTGGTGTCAGTTGTGTGTGTG | 250 | 0,5 | 3 | 55 |
|  | 6 | TGACACCAACACCTGCTTTC | TTTAGACGAGAGGGGAGACG | 163 | 0,5 | 2,5 | 58 |
|  | 7(1) | CGTCCTTAGCACGTGTTGC | GGTACTCGGGGGAGAGAGAT | 205 | 0,5 | 2,5 | 58 |
|  | 7(2) | GCTCGTCTTTGCAGAGTGGT | GGCACTGTTTGTCAGAGAAATG | 240 | 0,5 | 2,5 | 58 |
| ***MYL3*** | 1 | CCCTGCTTTCTGCATTCTTC | TGCCTCTTGCTAGGTCCACT | 258 | 0,5 | 2,5 | 55 |
|  | 2 | GGACAGGCTGAGACAGTTGAC | CAACCCCTGGGTTCAAGAC | 164 | 0,5 | 2 | 55 |
|  | 3 | GTAGGCTGACCCTGGGAGT | TAACACTATGGGGGCTCTCG | 232 | 0,5 | 2 | 56 |
|  | 4(1) | AGCCTTAGACCCTGGAACCT | ATTGCCCTCCTTGTCGAAG | 166 | 0,5 | 2 | 55 |
|  | 4(2) | CCAGCACATTTCCAAGAACA | GGCAACAGAGTGGTTTCTCC | 196 | 0,5 | 2 | 56 |
|  | 5 | GGAGAGGTTGAGCCACCAT | CTCCCCTCCCAGAAGACC | 214 | 0,5 | 2 | 56 |
|  | 6 | TCCCCATGTTGCACTCCT | GAGGCAGCAGGATGTCAAGT | 172 | 0,5 | 2,5 | 58 |
| ***MYLK2*** | 2(1) | TCCTGATGGGTGTCTCACCT | GGGCCTTGGCTGCTAGTT | 204 | 0,5 | 2,5 | 55 |
|  | 2(2) | TGGCCCAACCCTCAACTA | CCTCTGCTGCCTTCTTGC | 207 | 0,5 | 2,5 | 57 |
|  | 2(3) | AGACTGCGACACCTGAGACC | CACTCCCAGGAGGGGATATT | 198 | 0,5 | 2,5 | 56 |
| ***MYO6*** | 9 | TTTTCCCCTTTATTTGGTGA | CTTCAGAAGCACCAGCACAC | 260 | 0,5 | 2,5 | 55 |
| ***MYOZ2*** | 3* | ACGTTGGATGTGGGCAAAAAGGTCAGCATC | ACGTTGGATGTTAAATAGCCTGGCACCACG | 102 | 0,5 | 2,5 | 56 |
|  | 6* | ACGTTGGATGGGCAGACGGTCCTTTAATAG | ACGTTGGATGGTGGTATCATCTGTAGGTTC | 109 | 0,5 | 2,5 | 55 |
| ***OBSCN**** | 50 | ACGTTGGATGTCCAGCGCCTCCACATTCTC | ACGTTGGATGATGGTCTGATCCTCCCTCTC | 93 | 0,5 | 2,5 | 56 |
| ***PLN*** | 1(1) | TCCAGGCTACCTAAAAGAAGACA | AGGCATTTCAATGGTTGAGG | 165 | 0,5 | 2,5 | 55 |
|  | 1(2) | TGGAATGCCTCAACAAGCAC | TAAGCTGATGTGGCAAGCTG | 151 | 0,5 | 2,5 | 55 |
| ***PRKAG2*** | 5* | ACGTTGGATGGCTTTCCCGATACTAAAAGA | ACGTTGGATGTTGCAGGTACAGATTTATG | 116 | 0,5 | 2,5 | 55 |
|  | 9 | CCGCCTCATTTTCTAATTGC | AACATAGCTTTCCCGATACTAAAAGA | 219 | 0,5 | 2,5 | 55 |
|  | 15 | TTAATTTTCACGTGTCCTGCTTT | TTCTACCTCCCACCCTTCCT | 185 | 0,5 | 2,5 | 55 |
| ***RAF1*** | 7* | ACGTTGGATGTCATCTGAAGGTTCCCTCTC | ACGTTGGATGTCAATCATCCTGCTGTCCAC | 108 | 0,5 | 2,5 | 55 |
| ***SLC25A4*** | 2(1) | TCTACCCTGCCTGTCCTCTG | GTCCTTGAAGGCGAAGTTGA | 211 | 0,5 | 2 | 55 |
|  | 2(2) | AGCTCTCAACTTCGCCTTCA | CCAGACCATGGAACTCACG | 218 | 0,5 | 2,5 | 55 |
|  | 2(3) | CTACCCGCTGGACTTTGCTA | CTTTCCACACCACCCTCCT | 248 | 0,3 | 2,5 | 56 |
| ***TCAP*** | 1* | ACGTTGGATGAGTGATCATGGCTACCTCAG | ACGTTGGATGTCAGATCCTTCCATTCTGCC | 112 | 0,5 | 2,5 | 58 |
|  | 2(1) | CTCCCCAGCTGCTCCCTGCATGA | CTCCTTGGTGGCGCCCATCTTG | 210 | 0,5 | 2,5 | 56 |
|  | 2(2) | TCTTCACCCCTGCCAAGAT | CAGCAGCTCCCCTCCACAGT | 306 | 0,5 | 2,5 | 56 |
|  | 2(3) | GTCCCAGGAAGCACAGAGAG | TTGCCCTCACCAGTACCTTT | 300 | 0,5 | 2,5 | 58 |
|  | 2(4) | GACCCTCAGCCCCAGGAG | TGCAACTCTGGCACATGATTGAG | 222 | 0,5 | 2,5 | 56 |
| ***TNNC1*** | 1 | GGGGCAGGGCTATTTAAGTC | CTGAGTGAGCCCCCAGTG | 147 | 0,5 | 2,5 | 56 |
|  | 2 | CTGTGGAATGGGTTGGTTTC | CCTGGGGTCCTCTTCTGATAC | 184 | 0,5 | 3 | 56 |
|  | 3 | CCAACCCCTGAACACAGAGT | CCACCTCATCGATCATCTCC | 155 | 0,5 | 3 | 57 |
|  | 4 | CTCTGTAACGGCCATGCTG | TCACGTGCTCACTTGTCAAA | 157 | 0,5 | 2,5 | 57 |
|  | 5 | CTCCCCACAGAAATGCTGAT | CCACCCGCTTACCATCATAG | 159 | 0,5 | 2,5 | 55 |
|  | 6 | GCTGATCTCCTGCCTCCAT | GGGACACCAGAGCCAGATAG | 301 | 0,5 | 2,5 | 55 |
| ***TNNI3*** | 1 | GTCTGTGTCCTCGCCCTTTA | CCAACTCCCACTGCCTTG | 209 | 0,5 | 2,5 | 55 |
|  | 2 | TCCCCCGACCTCTTGTTCA | CAGACCCCTCACTGCAGCGC | 178 | 0,5 | 2,5 | 55 |
|  | 3 | CGGTAAGGGCTGGGTGGGGT | CCCTCCGGCGCCTGTACTCT | 184 | 0,5 | 2,5 | 55 |
|  | 4 | TGATCCTTCCTTGCTCCATC | CTGCCTGCTCTTTCCCAGT | 108 | 0,5 | 3 | 56 |
|  | 5 | CGCCTGGTCTTTATCCTGAA | TAGAAACCTCGCATCCTTGG | 221 | 0,5 | 2,5 | 56 |
|  | 6 | TTCCCCCAACAACACACAC | GTCAGGCAGAGACCAAGTCC | 195 | 0,5 | 2,5 | 55 |
|  | 7 | GTCAGGCAGAGACCAAGTCC | CCTCTTTCCTGGCCTTAGC | 250 | 0,5 | 3 | 55 |
|  | 8 | GACCCTAACCTCTGACTCATCG | CTGCCTAAGCCCTGGGTAA | 218 | 0,5 | 2,5 | 55 |
| ***TNNT2*** | 1 | GCTTAAAGCCCTCTCCATCC | CATCTCCCCGTCCATTCTC | 146 | 0,5 | 2,5 | 56 |
|  | 2 | GCTCATGAGGGGTGGAACTA | ACTCAGGCAAGATGCTCCA | 208 | 0,5 | 2,5 | 57 |
|  | 3 | AGGGAAAAGAAAGGGGGATT | CCAGGCAGCAAGAGAAGAGA | 215 | 0,5 | 2,5 | 57 |
|  | 4 | TGTGTTTGCATGTGCTTGTGTG | GCTCCCAGGATTTCCACATTGC | 250 | 0,5 | 2,5 | 55 |
|  | 5 | GGCTCTCTGCTCCCAGACTA | GAGGAAACGACTGACCCACT | 232 | 0,5 | 2,5 | 58 |
|  | 6 | TCTGGGTTCTGCCTGATAGC | ACATGGGAAAGCCTGTTCTG | 233 | 0,5 | 2,5 | 55 |
|  | 7 | CCCCAAAAACCAACAGAGTG | ATTCTCCTCCAAAGCTGCTG | 227 | 0,5 | 3 | 56 |
|  | 8 | GGAAATCCACAGGGATCTAGC | CGTGTCCACTGCACCATACT | 219 | 0,5 | 2,5 | 57 |
|  | 9 | CTATCATTGCTGTGGTTGGTCGG | CCAGCCCAAGGTCACAAAATCTC | 280 | 0,5 | 2,5 | 55 |
|  | 10 | CCCATCTCTCCTCTGGACTCT | CTGGGCCATCAGAGAATGTT | 221 | 0,5 | 2,5 | 55 |
|  | 11 | TGTCACCTTCTCCCTATGCAC | TGACTGATTTCATTCATGTTGATG | 231 | 0,5 | 2,5 | 55 |
|  | 12 | ATCAGGGTTTCCAATCCTTTC | GCAGTGGACACCTCATTCCT | 236 | 0,5 | 2,5 | 58 |
|  | 13 | TCTGGCCAGTTTACTCTGCTT | CAGTCCTGCCCTCTGGTG | 247 | 0,5 | 2,5 | 58 |
|  | 14 | CAGGAGGGCCCTTTCTTACT | CCCAGTGAACCAGGAGGAG | 204 | 0,2 | 2,5 | 56 |
|  | 15 | CTCCTTCTCCTCCTGCACTGC | ATGGGATAGCTGGAAGGTAGGG | 227 | 0,5 | 2,5 | 55 |
|  | 16(1) | GCCGCATGGTGACCTACTAC | CCCCATTTCCAAACAGGAG | 224 | 0,5 | 3 | 55 |
|  | 16(2) | CAGCTCCTGTTTGGAAATGG | AGGGTCTCGCCTCAGTTTCT | 158 | 0,5 | 2,5 | 55 |
| ***TPM1*** | 2 | TCCCTGTCCTTCTGGTTCTG | GGTGAGGGAAGCAGTGTGA | 257 | 0,5 | 2,5 | 56 |
|  | 3 | TCTCCCCAACTCTGAAATGC | AGCTGCAAAAGATGGCTGAT | 205 | 0,5 | 2,5 | 55 |
|  | 4 | TGTGCATTTGGGAAGTTCAG | ACTGCTGGGTGTCCACAAG | 218 | 0,5 | 2,5 | 55 |
|  | 5* | ACGTTGGATGGGTGTGTGTGTTGTGTCTTC | ACGTTGGATGTAGTCACTGCTCTGCAGCAC | 175 | 0,5 | 2,5 | 55 |
|  | 6 | TGATTTTGTGAATGGCCTTG | TGAAGTGAAGGTGCAGTCGT | 224 | 0,5 | 2,5 | 56 |
|  | 7 | GCCTGACATCTGGAATGCTC | CCGGTTCCATGAAAACAAAC | 159 | 0,5 | 2,5 | 56 |
|  | 8 | GTGATGTGCTTCATTTTCATCC | AATTGTTTTGCCAGGTTGGT | 230 | 0,5 | 2 | 56 |
|  | 9 | GCACCTCTGCCTTCCACTT | TGGAGATTAGGGAGCAAGGA | 200 | 0,5 | 2,5 | 58 |
| ***VCL*** | 7 | ACGTTGGATGGAGCATTGGCCTCCATAGAC | ACGTTGGATGCAGAACTAGAGGGTTACCTG | 108 | 0,5 | 2,5 | 55 |

1. Ho CY: **Hypertrophic cardiomyopathy**. *Heart failure clinics* 2010, **6**(2):141-159.
